# The longitudinal development of intrinsic timescales in infancy and their relation to alpha brain rhythm

**DOI:** 10.1093/cercor/bhag077

**Published:** 2026-06-24

**Authors:** Anna Truzzi, Josué Rico-Picó, Maria Rosario Rueda, Rhodri Cusack

**Affiliations:** School of Psychology, Queen’s University Belfast, David Keir Building, 18-30 Malone Road, BELFAST, BT9 5BN, United Kingdom; Department of Biobehavioural Sciences, Teachers College, Columbia University, Building 528, 525 W 120th St Suite 1159, New York, NY 10027, United States; Mind, Brain and Behaviour Research Center, University of Granada, Campus de Cartuja, s/n 18071 Granada, Spain; Department of Experimental Psychology, University of Granada, Campus Universitario de Cartuja, s/n18071 Granada, Spain; School of Psychology, Trinity College Dublin, Pearse St, Dublin, 2, Ireland; Trinity College Institute of Neuroscience, Trinity College Dublin, Dublin, Ireland

**Keywords:** brain timescales, developmental trajectory, infant, longitudinal, oscillatory, resting state EEG

## Abstract

Adult brain regions differ in the intrinsic timescales (INT) over which they integrate information. This spatial organization appears to emerge gradually: infants’ brain activity recorded during sleep with functional magnetic resonance imaging (fMRI) shows overall longer INT and a different spatial structure. However, since fMRI is sensitive to hemodynamic confounds and is affected by arousal state, these factors may have accounted for observed age-related differences. Here, we used electroencephalography (EEG) to investigate for the first time how INT develop in infancy in a longitudinal sample from 6 to 16-months-old (exploratory cohort, *n* = 45; validation cohort, *n* = 45) and adults (*n* = 10). Infants were awake and engaged in baseline visual protocol, and adults were recorded under comparable (and distinct) conditions. Infant intrinsic timescales shortened from 6 to 16-months but remained longer than those of adults at all ages. Finally, INT correlated with alpha lagged coherence, a metric of self-predictability and, to lesser extent, with alpha peak frequency, suggesting that alpha oscillatory activity may contribute to the emergence of INT. Identifying the mechanisms underlying longer INT early in infancy—a finding replicated across fMRI and EEG—is a crucial step toward understanding the neural computations that allow infants to extract and learn patterns from their environment.

## Introduction

Integrating information across multiple timescales is critical for constructing meaning from events distributed over time (Zacks and Tversky 2001). Computational models suggest that the brain’s intrinsic timescales (INT) index the temporal window over which information is integrated (Chaudhuri et al. 2015; Chang et al. 2025). In each region, INT reflect how long a time series stays similar to itself and, in adults and other mammals, it follows a cortical gradient—from short in sensorimotor areas to long in associative regions—that is consistently observed across measurement modalities with different temporal resolutions (EEG, fMRI, ECoG) and species (Hasson et al. 2008; Murray et al. 2014; Ito et al. 2020). Notably, EEG- and fMRI-based INT estimates are strongly correlated in adolescents (Watanabe et al. 2019). A growing body of evidence is highlighting that INT carry important information on the functional state of the brain (Wu and Gollo 2025) and that they are related to cognition, clinical symptoms, and individual variability (Honey et al. 2012; Liégeois et al. 2019; Golesorkhi et al. 2021). For example, in adolescents, more severe autism spectrum disorder (ASD) symptoms are associated with shorter INT in bilateral postcentral gyri and the right inferior occipital gyrus (Watanabe et al. 2019).

In neonates, INT distributions differ markedly from adults. Neonates show overall longer timescales measured with fMRI, though recordings were obtained during sleep (Truzzi and Cusack 2023). Naturalistic movie-viewing paradigms similarly report longer and less hierarchical event segmentation in infants compared to adults (Yates et al. 2022). The presence of structured timescales early in life suggests INT may serve as an inductive bias for learning slowly changing environmental features, supporting holistic representation and abstract reasoning.

However, previous fMRI-based infant studies face key limitations. First, fMRI measures hemodynamic rather than neural activity, and the infant hemodynamic response is slower and differs from that of adults (Arichi 2012). Thus, longer apparent INT might partly reflect vascular rather than neural difference and fMRI cannot capture timescales faster than ~1 Hz. Second, although INT are scale-invariant in adults across methods, this correspondence may not hold in infancy. Third, prior studies compared sleeping infants with awake adults, making arousal a major confound (Camacho et al. 2020). Fourth, the developmental trajectory of INT beyond the neonatal period remains unknown.

To address these limitations, we used resting state EEG (rs-EEG) in a large longitudinal cohort of awake infants (6, 9, and 16 months). EEG provides higher temporal resolution than fMRI and avoids confounds related to hemodynamic fluctuations and sleep–wake mismatches. We then compared infant INT with those of adults recorded under three resting conditions (video viewing, eyes closed, and eyes open) to characterize age-related changes.

In infants, we also investigated the relationship between INT and alpha-band properties, which show profound reconfiguration during infancy and toddlerhood (Anderson and Perone 2018; Rico-Picó et al. 2023; Wilkinson et al. 2023). We focused on the alpha band (adult, 9 to 11 Hz; infant, 6 to 9 Hz) due to its substantial development in this period and its key role in perceptual and attentional processes (Grandy et al. 2013; Haegens et al. 2014; Clayton et al. 2018). Alpha activity is also sensitive to neurodevelopmental conditions (Murphy et al. 2014; Keehn et al. 2017; Canigueral et al. 2022).

Since both INT and alpha oscillations have been related to temporal integration windows (Honey et al. 2012; Samaha and Postle 2015; Clayton et al. 2018), alpha oscillations are a plausible neural mechanism underlying age-related changes in INT.

Two alpha-related parameters may be associated with INT in infancy. First, alpha peak frequency increases with age (Marshall et al. 2002*;*  Schaworonkow and Voytek 2021; Rico-Picó et al. 2023) and is linked to faster segmentation of visual information (Freschl et al. 2019, 2021, 2022). Because faster alpha oscillations support shorter temporal integration windows (Samaha and Postle 2015, Clayton et al. 2018), we expected a negative correlation between alpha peak frequency and INT. Second, rhythmicity—or self-predictability—of the alpha signal also changes with age (Rayson et al. 2022). Lagged coherence measures the consistency of phase variation across time lags (Fransen et al. 2015). While most frequency bands show predictability only at short lags, alpha exhibits systematic variation over multiple cycles, making it a strong candidate rhythm associated with INT, which similarly reflects temporal self-similarity.

This study is the first to examine INT development across three infant time points using EEG, and the first to explore potential neural mechanisms underlying the emergence of INT. The primary aim was to characterize developmental changes in INT in infancy and early childhood, examine whether in this age range INT are comparable with adults’ INT values and spatial organization, and their relation to properties of the alpha brain rhythm. Understanding the typical development of INT and the underlying neural mechanisms provide a foundation for future research investigating whether longer early-life INT support learning of holistic and abstract representations and whether INT development diverges in neurodivergent populations.

## Materials and methods

### Participants

**Figure 1:**
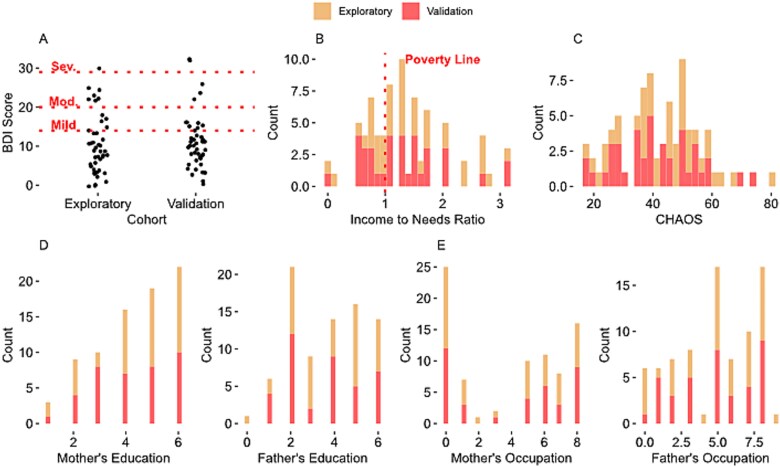
Descriptive graphics of home environment per cohort. In each panel, data are presented separately for two cohorts, exploratory and validation (*n* = 45 each), that were randomly selected while controlling for birth weight, gestational age, number of epochs, and sex. All analyses were first conducted in the exploratory cohort and then repeated in the validation cohort to provide an internal replication of the findings. The colors indicate the cohort (exploratory vs. validation). The top row displays BDI-II scores A) signaling its severity. Red dashed horizontal lines denote the thresholds for mild, moderate, and severe depressive symptoms. It also shows the CHAOS B) questionnaire and income to need punctuations per cohort C). The bottom row shows the number of mothers (pink) and fathers (beige) in the CNO-11 classification D) or educative level E). Notice that in occupation level, the scale is inverted with respect to CNO-11 (ie higher scores indicate more qualified jobs). Details are reported in the Supplementary Materials.

Infants were recruited from nurseries and hospitals in metropolitan Granada, Spain. Parents were approached in maternity wards and, if potentially interested in participating, were contacted again when their child was 5 months old. At the first visit, 160 infants were enrolled. Eighteen were excluded due to prematurity (<36 weeks), low birth weight (<2.7 kg), or family history of neurodevelopmental disorders (*n* = 4). Of the remaining sample, 123 attended the 9-month visit and 93 the 16 to 18-months visit. Analyses included children with at least one visit providing valid EEG data and sufficient epochs, allowing two missing time points and the analysis sample (*n* = 90). It was divided in two pseudo-random cohorts, exploratory and validation (*n* = 45 each), controlling for birth weight, gestational age, number of epochs, and sex. All analyses were first performed for the first cohort (exploratory) and repeated for the second cohort (validation) for validation purposes. The validation cohort did not differ from the exploratory cohort in other sociodemographic variables (all *P*s > 0.23) (Fig. 1, Table 1). Details on socioeconomic and environmental variables, as well as missing data distributions, are provided in the Supplementary Materials SI, Table S1, and Fig. S2. To provide an adult comparison group, we recruited 10 university students (*Mean* age = 22.0, *Standard Deviation (SD)* = 2.91), all Spanish descent and studying Education or Psychology. Participation was compensated with course credit. Both studies were approved by the Ethics Committee of the University of Granada (ref.: 488/CEIH/2018) and informed consent was obtained from all participants or legal guardians.

**Table 1:** Basic demographic information (mean and standard deviation) of the sample included in the exploratory and validation cohorts. For each cohort and time point (6, 9, and 16 months of age), the numbers of males and females in the sample are reported together with the average birth weight (BW), gestational age (GA) in weeks, age in days, and number of usable epochs provided by each infant.

Cohort	Visit	Sex	*N*	BW (grams)	GA (weeks)	Age (days)	Number of epochs
**Exploratory**	*6-mo.*	*F*	23	3,302.86 (388.06)	39.43 (1.63)	193.95 (8.64)	4.42 (2.34)
		*M*	20	3,368.33 (461.48)	39.61 (1.5)	192.7 (8.37)	5.82 (3.7)
	*9-mo.*	*F*	19	…	…	287.89 (11.72)	7.21 (3.34)
		*M*	18	…	…	284.89 (10.01)	7.5 (3.11)
	*16-mo.*	*F*	13	…	…	519.46 (25.32)	6.92 (3.58)
		*M*	23	…	…	516.56 (29.76)	10.22 (5.04)
**Validation**	*6-mo.*	*F*	22	3,229.75 (416.27)	39.15 (1.14)	196.23 (9.27)	6.31 (4.64)
		*M*	17	3,444.38 (703.35)	39.5 (1.55)	193.53 (9.52)	4.67 (3.03)
	*9-mo.*	*F*	18	…	…	284.53 (12.44)	8.72 (3.63)
		*M*	19	…	…	284.32 (9.1)	7.79 (4.34)
	*16-mo.*	*F*	14	…	…	516.21 (23.03)	8.14 (4.11)
		*M*	7	…	…	523.57 (21.62)	7.86 (5.4)

Note: BW, birth weight; GA, gestational age.

### Protocol

This study formed part of a larger longitudinal project examining brain function (rs-EEG) and cognitive maturation. Infants were tested at 6, 9, and 16 months with a consistent resting protocol, while other behavioral and EEG tasks varied. Sessions lasted ~30 min (6 months) or ~1 h (9, 16 months), with rs-EEG always performed last. Resting EEG comprised two 2-min blocks separated by a short break: (i) bubbles blown by the researcher; (ii) a video of geometric shapes with soft music. Since previous research has shown that the developmental trajectory of EEG signal is comparable across these two block types, we combined the block types to maximize generalizability across stimulus contexts and increase statistical power (see Supplementary Materials Figs. S10 and S11 in Rico-Picó and colleagues 2023).

Infants sat on their parents’ lap; parents were instructed not to interact. Gaze was not tracked. If the infant was very distressed and could not be soothed, EEG recording was stopped. Adult participants completed two EEG paradigms: an event-related protocol and rs-EEG. Resting EEG consisted of seven blocks: six alternating 1-min eyes-closed/eyes-open periods (fixation on a central box during eyes open), followed by the same video shown to infants.

### Electroencephalography processing

EEG was recorded using a 129-channel high-density geodesic net (EGI Geodesic Sensor Net, Eugene, OR, USA). The resting protocol was programmed in E-Prime 2.0 and synchronized with Net Station. Signals were digitized at 1,000 Hz, filtered online with 0.1 Hz high-pass and 100 Hz low-pass elliptical filters, and re-referenced to Cz. Infant sessions were videotaped, and periods of fussiness, parental interaction (eg signaling bubbles; St. John et al. 2016), or excessive movement were marked as invalid and excluded during segmentation. Preprocessing combined the Maryland Analysis of Developmental EEG (MADE) (Debnath et al. 2020) and Automated Pipeline for Infants Continuous EEG (APICE) (Fló et al. 2022) pipelines in EEGLAB (Delorme and Makeig 2004) using MATLAB 2021a. Following MADE, boundary electrodes were removed, data were filtered with a 0.2 to 30 Hz FIR Hamming filter, and noisy channels were identified with FASTER and removed. The MADE ICA procedure was then used to detect and remove ocular components. APICE was subsequently applied to detect artifacts based on power, amplitude, transitivity, and variance. Transient bad segments (<100 ms) were corrected via PCA (removing components explaining up to 90% variance). Data were segmented into 10 s non-overlapping epochs (12 per block for infants/adult video; 6 per block for adult eyes-open/eyes-closed). Epochs with >30% noisy data were discarded; otherwise, bad electrodes, if any, were interpolated. Global bad channels were reintroduced by spherical interpolation, and data were re-referenced to the average. A trained researcher performed a final visual inspection, removing remaining noisy epochs. A linear mixed model (random intercept per participant) indicated trial numbers increased with age in both cohorts (Exploratory: B = 1.23, *P* < 0.001; Validation: B = 1.51, *P* < 0.001), with significant negative quadratic effects (Exploratory: B = −0.09, *P* = 0.012; Validation: B = −0.11, *P* = 0.008). Thus, number of trials was included as a covariate in further analysis.

### Electroencephalography parameters

We computed three metrics of interest for the current study: INT, alpha peak frequency extracted from the oscillatory power-spectrum, and lagged coherence. Lagged coherence and INT were individually computed by epoch and electrode and then averaged across epochs. In contrast, alpha peak frequency results from the averaged power-spectrum across epochs computed individually per electrode. For analysis controlling whether there were regional differences, electrodes’ individual values were grouped into five clusters (temporal, occipital, parietal, frontal, and central, Fig. S1).

#### Intrinsic timescales

Intrinsic timescales were computed using Python 3.8 and the *scipy* library (*scipy.signal.hilbert*). For each epoch, we calculated the autocorrelation function on the envelope of the EEG signal using the Hilbert transformation (Watanabe et al. 2019). To favor the comparison with our previous work on fMRI, for each electrode, we fitted the autocorrelation function with an exponential curve (*scipy.optimize.curve_fit*) and calculated Tau ($\tau$) as its decay coefficient (Ito et al. 2020), with the constraint of a positive scaling factor (A), given the biological implausibility of negative values for this variable, and a positive decay coefficient $\tau$. This formulation assumes that the autocorrelation of the signal decays by an equal factor (1/e) in each successive time period, and thus follows an exponential decay $\tau,$ a common approximation for the temporal integration in neural time series. Therefore, we used an exponential fit, using the following form:


(1) \begin{eqnarray*} R(k)=A\left({e}^{\frac{-k\Delta }{\tau }}+B\right) \end{eqnarray*}

Where *R* is the autocorrelation value at temporal lag *k* and *A* the scaling value. $\Delta$ is the sample period, $\tau$ the intrinsic timescale, where $\tau$ represents the intrinsic timescale, ie the rate at which the autocorrelation decays over time, with larger $\tau$ values indicating slower decay and, therefore, longer temporal dependencies in the signal, and *B* the asymptotic autocorrelation level. A window of 1,000 lags and the Trust Region Reflective optimization (Branch et al. 1999) algorithm were used to fit the decay curves with max iterations set to 2,000. Further details on model fit and missing values are available in the supplementary materials (Section number: SII, Tables S2–S4, Fig. S2–S5) and a visualization of how the exponential fit models the autocorrelation function in short vs. long $\tau$.

However, since previous research has calculated the INT from EEG signals by calculating the area under the autocorrelation function (Watanabe et al. 2019; Tang et al. 2025), we also estimated intrinsic timescales in this same way (INT_AUC) and compared it with the INT estimated on the fitted exponential curve. The INT and INT_AUC values were generally strongly correlated (0.74 < *r* < 0.89) but for the 6 months exploratory cohort (*r* = 0.57) (see Fig. S6). Therefore, analysis was also repeated on the INT_AUC values and are reported here when they differ from the main analysis.

#### Alpha-band oscillatory parameters and rhythmicity

Given the impact of the aperiodic part of the signal on absolute brain activity (Donoghue et al. 2020a; Voytek and Knight 2015), we isolated the oscillatory component using the *Specparam* toolbox (Donoghue et al. 2020b) using an EEGLAB wrap-up. This toolbox decomposes the power-spectrum obtained by *spectopo* function into oscillatory and aperiodic components. It considers the power at each frequency as a combination of aperiodic and oscillatory activity (P(*f*)). The aperiodic components (*L*) correspond to an initial offset (ie power at the minimum frequency), combined with a decaying exponent that creates a decaying background curve.


(2) \begin{eqnarray*} L(f)=b-\log (fx) \end{eqnarray*}

where aperiodic component *L* corresponds to the power at each frequency, *b* is the offset value of the power, and *x* is the aperiodic exponent. The oscillatory power is defined as follows:


(3) \begin{eqnarray*} {G}_n=a{e}^{-\frac{{\left(F-c\right)}^2}{(2w)^2}} \end{eqnarray*}

where *a* is the amplitude of the peak, *c is* the center frequency in Hz, and *w* represents the standard deviation of the Gaussian curve. Therefore, the absolute power is a combination of *L* at a given frequency and the oscillatory curve above it.

EEG power parametrization parameters were based on previous infant studies (peak width limits: [2.5 to 12 Hz], maximum number of peaks: 5, aperiodic mode: fixed, peak threshold: 2) with 0.1 Hz steps resolution in the FFT. We examined 1 to 20 Hz range as our focus was the alpha band and shorter ranges improve the parametrization fit in pediatric populations (Schaworonkow and Voytek 2021; Rico-Picó et al. 2023). Power-spectrum parametrization fit of the models proved to be appropriate independently of the cohort and the visit (Exploratory: 6-months R2 = 0.991 (0.003), 9-months R2 = 0.991 (0.003), 16-months R2 = 0.993 (0.003); Validation: 6-months R2 = 0.989 (0.003), 9-months R2 = 0.991 (0.004), 16-months R2 = 0.993 (0.003)). Furthermore, the error between the absolute and parametrized power-spectrum was minimal in the alpha band, while the oscillatory power spectrum presented a marked alpha peak in the expected range (see Supplementary Fig. S7).

Once the oscillatory part of the power spectrum was computed, we extracted the oscillatory parameters provided by the *Specparam* toolbox. Given the expected age-related increase in alpha peak frequency (Marshall et al. 2002; Freschl et al. 2022; Rico-Picó et al. 2023), we adjusted the alpha band for each infant considering the average individual alpha peak frequency (IAF) in *specparam* oscillatory peaks that occurred between 5.5 and 8 Hz over the parieto-occipital electrodes (see Freschl et al. 2022, Rico-Picó et al. 2023). IAF was then used to compute the alpha range as IAF +– Bandwidth/2, which was then used to compute alpha power and set the alpha band in the lagged coherence metric. Next, we conducted an exploratory analysis of oscillatory power by subtracting the aperiodic background curve from the absolute power and extracting the energy in the alpha band. Notice that even if we extracted IAF from parieto-occipital regions to obtain the individual frequency for the alpha bandwidth computation, for analysis IAF and oscillatory power were extracted from all the electrodes to be consistent with INT computation.

To explore the rhythmic properties in relation to the INT, we followed the guide proposed by Rayson et al. (2022) employing Fieldtrip v2018a (Oostenveld et al. 2011). We focused on lagged coherence as a measure of signal rhythmicity (Fransen et al. 2015). This metric evaluates the predictability of the signal of each electrode over different time lags. To this end, the signal is decomposed, and the phases of the signal are extracted. Subsequently, the differences in phase between adjacent time points are computed varying in their temporal distance (lags), which is normalized by the differences in all adjacent time points. Thus, larger values in lagged coherence represent a systemic variation during the same time lag, which represents a rhythmic pattern (ie self-predictable). We computed lagged coherence from 2 to 20 Hz, from 1.5 up to 5 lags, in 0.1 lag steps. However, for the current analysis, we extracted lagged-coherence activity in the alpha band provided by *specparam* toolbox (ie IAF). Lagged coherence between 1.5 to 2 lags was considered as burst predictability, while the values between 2.5 and 4 lags corresponded with the rhythmic predictability.

### Analysis

#### Longitudinal analysis

We employed linear mixed models (LMM) in R using the lme4 package (Bates et al. 2014) to unveil the trajectories of INT $\tau$ values. The models were constructed using a bottom-up approach (West et al. 2006). We first fitted the random effects of the model (random intercept vs. random slope) and then added the fixed effects. First, we introduced Age, then Cluster, Time Squared, and finally their interactions. We selected the model that best fitted the corrected Akaike Information Criteria (cAIC), and we checked the final model based on the distribution of residuals and the collinearity of the variables. If the residuals were non-normally distributed or had high collinearity (VIF > 10), we transformed the data based on Tukey’s ladder of power or reduced the model, respectively. We computed the degrees of freedom using the Satterthwaite approximation and the effect size of the model based on Nakagawa et al. (2017) R^2^ approach. When the cluster main effect was significant, we performed pairwise analyses corrected using Hommel (Blakesley et al. 2009) to determine cluster differences in INT values. All analyses were controlled for percentage of non-convergent electrodes as a proxy of data quality. In Supplementary Section SIII (Tables S5–S7, Fig. S8), we also explored the robustness of the models by introducing sociodemographic variables of interest as covariates and equaling the number of trials for all the infants.

#### Comparison with adults

To investigate the differences between infants and adults, the distribution of the average infant INT values per electrode at each time point (6, 9, and 16 months) were compared with the average adult data for each condition (video, eyes open, and eyes closed) using a Kruskal–Wallis test. Mann–Whitney U tests were then run as post hoc tests and Cohen’s d was used to determine the effect size. Bonferroni correction was applied to account for multiple comparisons (α = 0.008).

While this approach investigated whether the overall average INT values differed between groups, it did not capture similarities or differences in the spatial distribution of INT. Specifically, while infants may have overall longer timescales compared to adults, they could still present a similar relative hierarchical pattern, albeit at higher absolute values. To investigate this, for each adult condition and infant age, a linear model was run to predict each infant’s spatial INT pattern from the average adult spatial pattern computed across participants within each condition. T-tests were used to assess whether the resulting distributions of beta values differed from zero and from each other.

#### Relationship between intrinsic timescales and properties of alpha activity

To determine whether INT and alpha-band oscillatory power and frequency were related, we followed two different strategies. First, we averaged alpha peak frequency, power, lagged coherence, and INT across epochs and participants, which resulted in a single average value per electrode. Secondly, we averaged alpha properties and INT across epochs and electrodes per participant, which resulted in a single average value per individual. Spearman’s rank correlation analysis was used to investigate whether the alpha spatial properties and/or individual differences were correlated to INT as an average individual feature, disregarding spatial patterns. All the correlations were controlled for the percentage of non-convergent electrodes. In addition, analysis including oscillatory related parameters (ie alpha peak frequency and alpha oscillatory power) were controlled for the fit of the models (R^2^) of the power spectrum decomposition. This analysis was only conducted in the developmental sample.

## Results

### Early development of the intrinsic timescales

**Figure 2:**
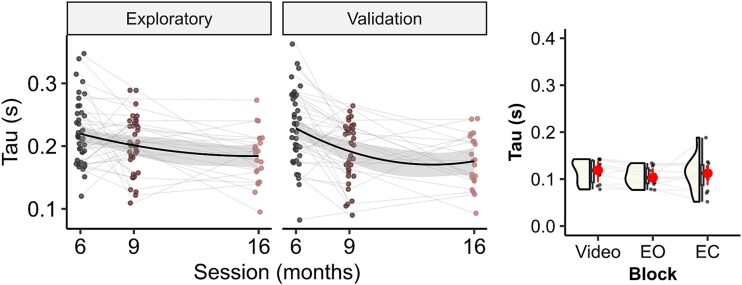
Intrinsic timescales development in the developmental and adult cohorts. Each dot corresponds to a participant INT values averaged across epochs and electrodes, the thin lines represent individual trajectories. For the exploratory and validation longitudinal infant cohorts, on the *X* axis the data are grouped by Age point (6, 9, and 16 months of age). For the adult cohort, on the *X* axis the data are grouped by condition (video, eyes open – EO, eyes closed – EC). The adult plots are shown nearby the longitudinal infant plots to allow for an easier visualization of the comparison between the cross-cohorts $\tau$ values.

**Figure 3:**
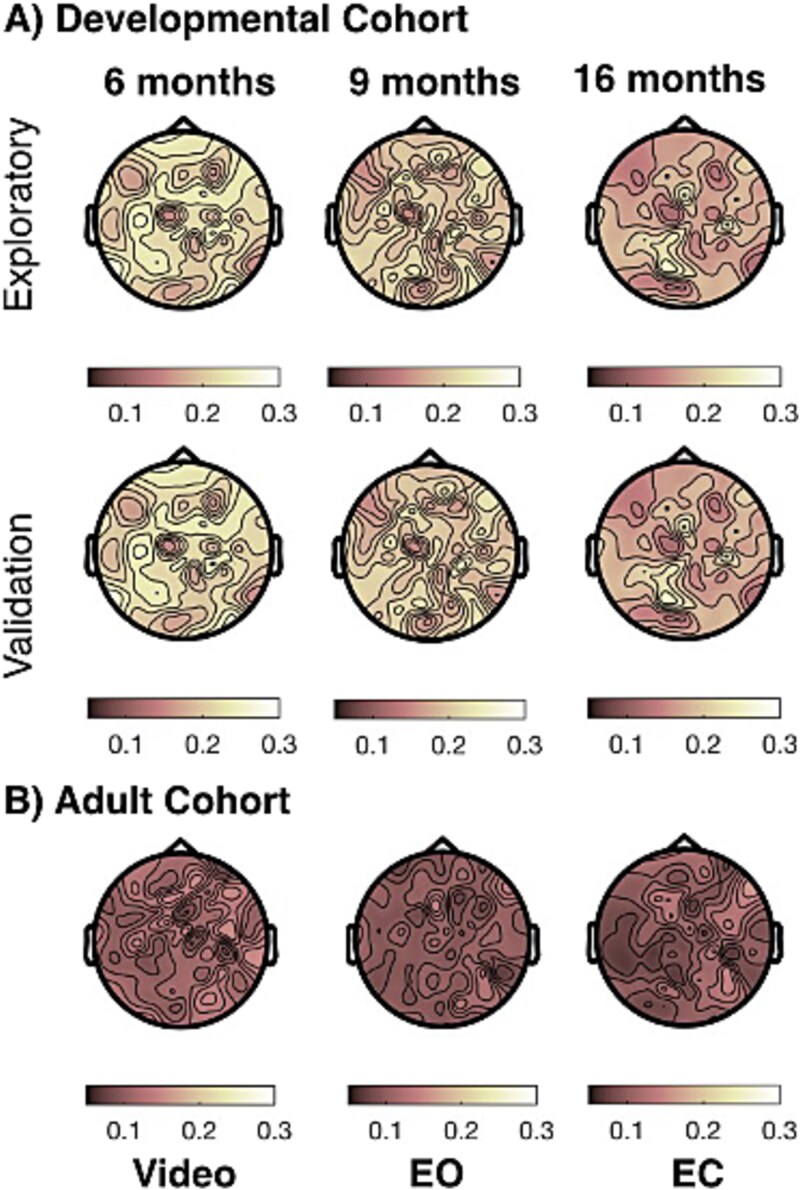
Topographical distribution of the average intrinsic timescales’ values in the developmental and adult cohorts by electrodes. Darker colors indicate shorter timescales; lighter colors indicate longer timescales. The first two rows show the data for both developmental cohorts: exploratory (first row) and validation (second row). The third row shows the data for the adult cohort collected in the three different conditions: video, eyes open (EO), and eyes closed (EC). The topographical distribution allows to visualize how similar vs different the spatial patterns of INT are between infants and adults and to compare the adults’ distribution of average INT across different conditions.

We evaluated the development of INT $\tau$ values per group using a linear mixed model analysis (Table 2). Descriptions of the average $\tau$ values and their distributions can be found in Table 3 and Figs. 2 and 3. Further details of INT per cluster in infants are provided in Supp. Tables S6 and S7 and Fig. S8. In the exploratory cohort, the best model included Age, Age Squared, and Cluster and Age x Cluster interaction as fixed factors with a random Age slope per participant. $\tau$ values (marginal *R^2^* = 0.18, conditional *R*^2^ = 0.32) were significantly reduced with age with a negative quadratic effect (Age: B = −0.11, *P* < 0.001; Age squared: B = 0.09, *P* < 0.001). Exploratory LMM considering each interval with random intercept per child revealed that $\tau$ values decreased between 6 and 9 (B = −0.07, *P =* 0*.*0254, 95% CI = [−0.13, −0.01]) and 9 and 16 months (B = −0.03, *P* = 0.009, 95% CI = [−0.06, −0.01]). $\tau$ reduction with age was less pronounced in the occipital cluster (B = 0.05, *P* < 0.001) compared to the central cluster and did not occur in the rest of studied clusters (all *P* > 0.23). The occipital cluster presented larger $\tau$ values than the central cluster (all *z* = 3.24, *P* = 0.02), but no other pairwise comparisons were significant after Holm correction (all *z*s < 2.86, all *p*s > 0.05).

**Table 2:** $\tau$ development in the exploratory and validation cohorts for age, age squared, and intercept fixed effects. Beta, standard error, CI, t values, and *P* values are reported for each parameter and cohort.

Cohort	Fixed effect	B	SE	CI	t	df	*P*
**Exploratory**	*Intercept*	0.18^***^	0.01	[0.17, 0.20]	22.93	68.92	**1.11e-64**
	*Age*	−0.11^***^	0.03	[−0.16, −0.06]	−4.35	138.87	**2.64e-5**
	*Age squared*	0.09^***^	0.02	[0.04, 0.14]	3.66	368.42	**2.85e-4**
**Validation**	*Intercept*	0.36^***^	0.01	[0.32, 0.43]	25.83	60.33	**3.68e-39**
	*Age*	−0.34^***^	0.05	[−0.43, −0.24]	−7.26	109.70	**5.76e-11**
	*Age squared*	0.29^***^	0.05	[0.19, 0.39]	5.90	367.23	**8.10e-09**

Note: CI were obtained with bootstrapping (iterations = 5,000). Significant *p* values are shown in bold. ^*^P < 0.008, ^**^P < 0.01, ^***^P < 0.001.

**Table 3:** $\tau$ values (mean, standard deviation) were calculated by visit and sex in the exploratory and validation cohorts. For adults, the $\mathrm{\tau}$ values (mean, standard deviation) are reported for each of the different cross-sectional conditions: video, eyes open (EO), eyes closed (EC).

Visit		*Cohort*	
		*Exploratory*	*Validation*
**Infants**	*6-mo*	0.21 (0.17)	0.21 (0.18)
	*9-mo*	0.19 (0.16)	0.18 (0.15)
	*16-mo*	0.18 (0.14)	0.17 (0.14)
	*Video*	0.12 (0.10)	…
**Adults**	*EO*	0.11 (0.001)	…
	***EC***	**0.10 (0.001)**	**…**

Note: Descriptive statistics were computed for participants who had valid data for each visit.

In the validation cohort, the best model included a random slope of Age per participant, Age, Age Squared, and Cluster as the fixed effects. $\tau$ values (marginal *R^2^* = 0.18, conditional *R*^2^ = 0.29) decreased across age (B = −0.18, *P* < 0.001) with a positive quadratic effect (B = 0.16, *P* < 0.001). However, when these intervals were examined individually, there was a significant change from 6 to 9 months (B = −0.15, *P* < 0.001, 95% CI = [−0.18, −0.12]) but no change from 9 to 16 months (B = −0.01, *P* = 0.435, 95% CI = [−0.02, 0.01]). The occipital cluster presented larger INT values than the rest of the clusters (all *z*s > 3.45, all *p*s < 0.01), except for the frontal pole cluster (*z =* 2.01*, P* = 0.34). No other cluster differences were significant (all *z*s < 2.67, all *p*s < 0.08). Results divided by cluster are presented in Figure S9. Therefore, $\tau$ values decreased with age independently of the cohort studied. However, Age x Region results were not robust, suggesting a reduction independent of electrode region. Importantly, both cohorts presented significant quadratic effect, which resulted in either smaller change or no change depending on the cohort.

Analysis run on the INT_AUC values lead to slightly different but compatible results. The exploratory cohort displayed a significant quadratic effect but a nonsignificant linear effect of age. Based on the significant quadratic effects, we decided to conduct a follow-up analysis testing the differences by pairs of visits. Showing that there were no significant differences between visits 6 and 9 months in terms of INT_AUC (B = −0.02, 95% CI = [−0.05, 0.00], *P* = 0.94), but 9 month visit values were larger than at 16 months (B = −0.03, 95% CI = [−0.05, −0.02], *P* < 0.001). In the validation cohort, the linear but not the quadratic effect was significant. Follow-up pairwise comparisons unveiled that the reduction was significant between both 6 to 9 months period (B = −0.10, 95% CI = [−0.16, −0.05], *P* < 0.001) and 9 to 16 months period (B = −0.04, 95% CI = [−0.06, −0.2], *P* = 0.020). Overall, results across estimation methods consistently indicate that intrinsic timescales decrease from 6 to 16 months.

### Comparison with adults

#### Overall distribution across electrodes

The distributions of brain timescales at different ages were significantly different from that of adults regardless of the adult condition they were compared with (adults video vs. infants at 6, 9, 16 mo.: Kruskal–Wallis *H* = 299.66, *P* < 0.001; adults eyes open vs. infants at 6, 9, 16 mo.: *H* = 302.30, *P* < 0.001; adults’ eyes closed vs. infants at 6, 9, 16 mo.: *H* = 301.07, *P* < 0.001). These differences were replicated in the validation group (adults video vs. infants 6, 9, 16 months: *H* = 302.09, *P* < 0.001; adults eyes open vs. infants at 6, 9, 16 mo.: *H* = 308.32, *P* < 0.001; adults’ eyes closed vs. infants at 6, 9, 16 mo.: *H* = 305.65, *P* < 0.001). Post hoc Mann–Whitney U tests confirmed that INT at all ages, 6, 9, and 16 months, were significantly longer compared to adults regardless of the condition adults were in (Table 4). Results were unchanged for the INT_AUC values (Table S8). There were significant differences also within the adult group and across conditions (Kruskal–Wallis H = 32.14, *P* < 0.001). Post hoc Mann–Whitney U tests (Bonferroni-corrected α = 0.008) revealed significant differences between some conditions. Specifically, INT were significantly longer during video watching compared to eyes-open (M_video = 0.12; M_eyes_open = 0.11; U = 8328, *P* < 0.001, d = 0.69) and eyes-closed (M_video = 0.12; M_eyes_closed = 0.11; U = 8105, *P* < 0.001, d = 0.67). INT in the eyes-open and eyes-closed conditions instead did not significantly differ.

**Table 4:** Comparison between $\mathrm{\tau}$ values’ distributions in infants and adults for the different visits and conditions. Mann–Whitney U statistics and Cohen’s d effect size are reported for each comparison. The $\tau$ values’ distributions comparisons were run separately for the infant exploratory and validation cohorts.

Infant visit	Adult condition	Exploratory		Validation	
		Mann–Whitney U	*d*	Mann–Whitney U	*d*
**6-mo.**	Video	11,879 ^***^	5.0	11,879^***^	4.5
	EO	11,880^***^	5.6	11,880^***^	5.1
	EC	11,877^***^	5.0	11,877^***^	4.7
**9-mo.**	Video	11,870^***^	4.4	11,867^***^	3.8
	EO	11,878^***^	5.1	11,876^***^	4.5
	EC	11,870^***^	4.5	11,856^***^	4.0
**16-mo.**	Video	11,658 ^***^	2.8	11,301^***^	2.4
	EO	11,785^***^	3.4	11,618^***^	2.9
	EC	11,737^***^	3.1	11,510 ^***^	2.7

^*^P < 0.008, ^**^P < 0.01, ^***^P < 0.001. EO = Eyes Open, EC = Eyes closed.

#### Predicting infant spatial patterns from adult data

The previous analysis allowed us to compare the average distribution of INT across groups and showed that infants have overall longer timescales compared to adults. However, averaging across electrodes removes the information about the spatial distribution of the intrinsic timescales, which might still retain similarity between infants and adults. Namely, infants might still present a relative hierarchical distribution of timescales similar to adults, albeit at higher absolute values. To assess the similarity of the INT spatial distribution in infants and adults, we used a linear regression model to predict individual infants’ spatial patterns from the average adult spatial pattern computed across participants within each condition: videos, eyes open, and eyes closed. The resulting beta distributions were compared, and no comparison was significant in both the exploratory and the validation cohort (Table 5). When replicating the analysis on the INT_AUC values, beta distributions were consistently significantly different from zero when infant intrinsic timescales were modeled from adult INT in the eyes open condition. However, the variance explained remained small with 0.02 < *R^2^* < 0.07 which indicate trivial predictive power (see Table S9 for the complete results). These results show that up to 16 months of age, not only the overall individual INT values are longer compared to adults but also the INT distribution across electrodes is not comparable with the adult distribution of INT.

**Table 5:** Mean and standard deviation of the betas distributions for the linear models predicting infant timescales spatial distribution starting from the average adult timescales in different conditions. Confidence interval (CI) and t values resulting from testing whether the distributions are significantly different from zero are reported.

Infant visit	Adult condition	Exploratory cohort				Validation cohort			
		*M(SD)*	*CI*	*t*	*R^2^*	*M(SD)*	*CI*	*t*	*R^2^*
**6-mo.**	*Video*	0.00 (1.07)	[−0.34, 0.34]	0.001	…	0.1 (1.24)	[0.31, 0.51]	0.5	…
	*EO*	−0.13 (1.15)	[−0.5, 0.23]	−0.73	…	0.19 (1.07)	[−0.16, 0.54]	1.11	…
	*EC*	0.07 (0.84)	[−0.19, 0.34]	0.55	…	0.13 (0.81)	[−0.14, 0.39]	0.98	…
**9-mo.**	*Video*	0.18 (0.7)	[−0.05, 0.42]	1.59	…	−0.10 (0.79)	[−0.37, 0.16]	−0.78	…
	*EO*	0.35 (0.80)	[0.08, 0.61]	2.6^*^	0.03	−0.012 (0.86)	[−0.30, 0.28]	−0.08^*^	…
	*EC*	0.21 (0.59)	[0.01, 0.41]	2.12	…	0.01 (0.72)	[−0.24, 0.25]	0.04	…
**16-mo.**	*Video*	0.08 (0.60)	[−0.2, 0.35]	0.6	…	−0.01 (0.56)	[−0.35, 0.17]	−0.8	…
	*EO*	0.30 (0.62)	[0.02, 0.58]	2.50	…	0.29 (0.52)	[0.05, 0.53]	2.5	…
	*EC*	0.08 (0.47)	[−0.13, 0.30]	0.84	…	−0.02 (0.42)	[−0.22, 0.17]	−0.24	…

^*^P < 0.008, ^**^P < 0.01, ^***^P < 0.001*.* EO = Eyes open, EC = Eyes closed.

### Alpha properties and intrinsic timescales

**Figure 4:**
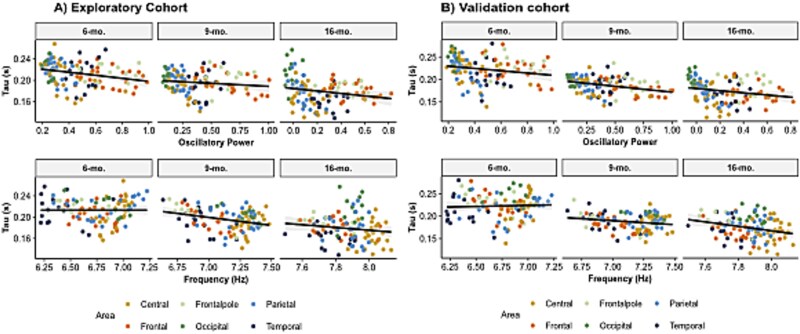
Spatial correlations between $\tau$ values and alpha parameters (oscillatory power and peak frequency) in the exploratory A) and validation B) cohorts. Each dot corresponds to an averaged $\tau$ value per electrode, and the shaded area represents the standard error. Each row stands for a session (6, 9, and 16 months). Where correlations are significant across INT estimation methods, the corresponding values are shown in the plots. ^*^P < 0.05, ^**^P < 0.01, ^***^P < 0.001.

**Figure 5:**
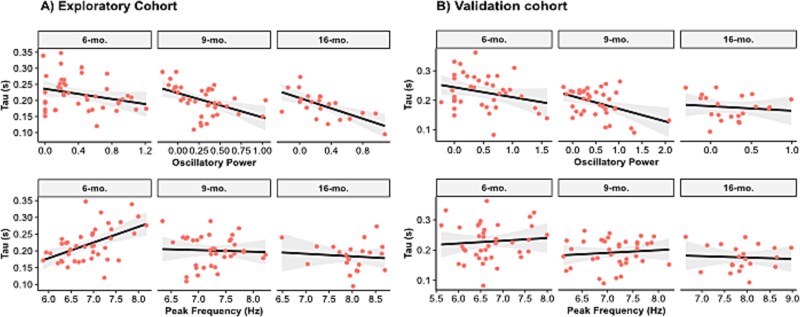
Individual correlations between individual participants’ all brain $\tau$ values average and alpha power (Osc. Power) and peak frequency in the exploratory A) and validation B) cohorts. Each dot corresponds to an individual participant, and the shaded area represents the standard error. Each row reports the correlations for a visit (6, 9, and 16 months). Each point was obtained by averaging across the electrodes of the clusters of interest. Where correlations are significant across INT estimation methods, the corresponding values are shown in the plots. ^*^P < 0.05, ^**^P < 0.01, ^***^P < 0.001.

**Figure 6:**
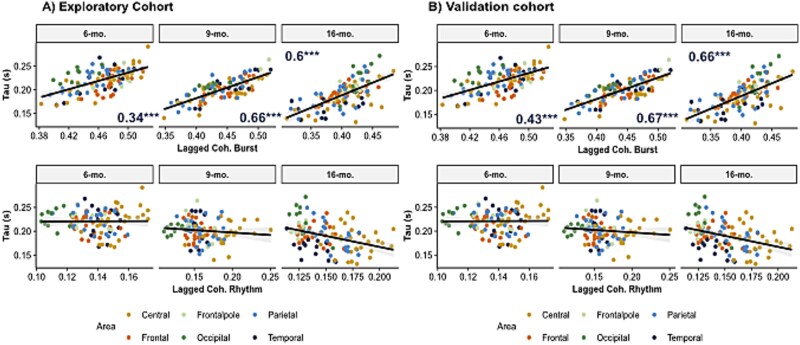
Spatial correlations between $\tau$ values and alpha lagged coherence parameters in the exploratory A) and validation B) cohorts. Each dot corresponds with the averaged $\tau$ values across participants per electrode, and the shaded area is the standard error. Each row stands for a session (6, 9, and 16 months). Where correlations are significant across INT estimation methods, the corresponding values are shown in the plots. ^*^P < 0.05, ^**^P < 0.01, ^***^P < 0.001.

**Figure 7:**
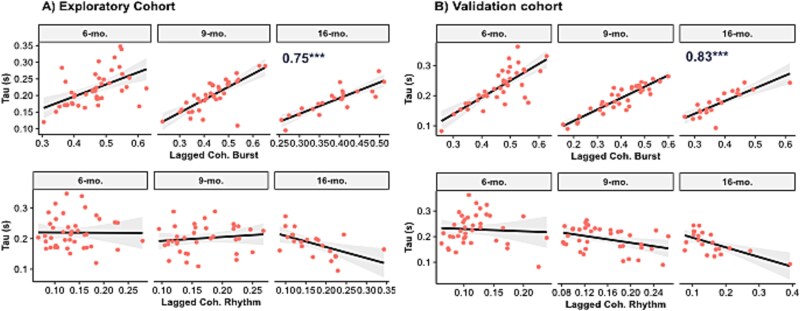
Individual correlations between individual participants’ all brain $\tau$ values average and alpha-lagged coherence parameters in the base A) and validation B) cohorts. Each dot corresponds to an individual participant, and the shaded area represents the standard error. Each column reports the correlations for a visit (6, 9, and 16 months). Each point was obtained by averaging across the electrodes of the clusters of interest. Where correlations are significant across INT estimation methods, the corresponding values are shown in the plots. ^*^P < 0.05, ^**^P < 0.01, ^***^P < 0.001.

We evaluated the relationship between alpha band properties and INT, considering both spatial distribution and individual differences in $\tau$ and alpha values. Here, we report only results that were significant in both the exploratory and validation cohorts for both the INT and INT_AUC values. For a full account of the findings, see Tables 6 and 7 and Figs 4 to 7. The descriptive information regarding alpha activity can be found in Tables S9 to 15. For the INT values of interest and Tables S16 and S17 for the INT_AUC results.

**Table 6:** Partial Spearman rank correlation between $\mathrm{\tau}$ values and alpha peak frequency and power controlling for percentage of non-convergent electrodes and fit of the models in the power spectrum decomposition. The correlation was run separately for each visit within the exploratory and validation independent samples. The table displays correlation between the alpha features and the average INT per electrode across participants, and individual’s INT across electrodes. This table reports results for the INT values estimated on the exponential curve fitted to the autocorrelation function. Results for the INT values estimated from the area under the curve see Table S16.

INT calculation	Visit	n	Exploratory		n	Validation	
			*Frequency*	*Power*		*Frequency*	*Power*
**INT per electrode (across individuals)**	*6-mo.*	…	−0.08 [−0.26–0.1]	−0.23^*^ [−0.39–−0.06]	…	−0.02 [−0.2–0.18]	−0.2 [−0.36–−0.01]
	*9-mo.*	…	−0.41^***^ [−0.56–−0.25]	−0.21^*^ [−0.37–−0.06]	…	−0.38^***^ [−0.52–−0.19]	−0.37^***^ [−0.51–−0.2]
	*16-mo.*	…	−0.23^*^ [−0.37–−0.08]	−0.11 [−0.24–0.02]	…	−0.39^***^ [−0.54–−0.22]	−0.16 [−0.33–0.02]
**INT per individual (across electrodes)**	*6-mo.*	43	0.49^**^ [0.19–0.7]	−0.26 [−0.54–0.08]	39	0.09 [−0.28–0.43]	−0.16 [−0.49–0.2]
	*9-mo.*	37	−0.05 [−0.36–0.29]	−0.44^*^ [−0.71–−0.1]	37	−0.11 [−0.44–0.26]	−0.45^*^ [−0.7–−0.11]
	*16-mo.*	22	−0.05 [−0.49–0.4]	−0.55^*^ [−0.84–0.26]	21	−0.01 [−0.55–0.53]	−0.31 [−0.79–0.26]

^*^ *P* < 0.05, ^**^P < 0.001, ^***^P < 0.001. Confidence intervals were computed by bootstrapping with replacement (5,000 iterations).

**Table 7:** Partial Spearman rank correlation between $\mathrm{\tau}$ values and burst and rhythmic properties of the alpha controlling for percentage of non-convergent electrodes. The correlation was run separately for each visit within the exploratory and validation independent samples. The table displays the correlation between the alpha features and the average INT across individuals per electrode (spatial distribution) and between the alpha features and each individual participant’s INT per electrode (individual differences). This table reports results for the INT values estimated on the exponential curve fitted to the autocorrelation function. Results for the INT values estimated from the area under the curve see Table S17. Bold statistics highlight which results were significant across INT estimation values.

Correlation Type	Visit	n	Exploratory		n	Validation	
			*Burst*	*Rhythm*		*Burst*	*Rhythm*
**INT per electrode (across individuals)**	*6-mo.*	…	0.34^***^ **[0.15–0.5]**	0.11 [−0.09–0.29]	…	0.43^***^ **[0.23–0.6]**	−0.11 [−0.31–0.09]
	*9-mo.*	…	0.66^***^ **[0.53–0.77]**	−0.5^***^ [−0.62–−0.36]	…	0.67^***^ **[0.55–0.77]**	−0.37^***^ [−0.54–−0.16]
	*16-mo.*	…	0.6^***^ **[0.44–0.73]**	−0.47^***^ [−0.61–−0.29]	…	0.66^***^ **[0.53–0.76]**	−0.48^***^ [−0.62–−0.31]
**INT per individual (across electrodes)**	*6-mo.*	43	0.38^*^ [0.07–0.65]	−0.22 [−0.48–0.1]	39	0.55^***^ [0.13–0.83]	0.08 [−0.24–0.39]
	*9-mo.*	37	0.36 [−0.19–0.78]	−0.32 [−0.59–0.1]	37	0.66^***^ [0.33–0.85]	−0.36^*^ [−0.61–−0.05]
	*16-mo.*	22	0.75^***^ **[0.37–0.9]**	−0.41 [−0.75–0.03]	21	0.83^***^ **[0.48–0.95]**	−0.57^*^ [−0.83–−0.2]

^*^ *P* < 0.05, ^**^P < 0.001, ^***^P < 0.001. Confidence intervals were computed by bootstrapping with replacement (5,000 iterations). Bold statistics highlight which results were significant across INT estimation values.

Alpha peak frequency and oscillatory power (Table 6; Figs. 4 and 5). No reliable significant relation was found between INT values and alpha peak frequency at the individual level or over space.

Alpha lag coherence burst and rhythm (Table 7; Figs. 6 and 7). Over space, INT length was significantly positively correlated with alpha lag coherence burst at all ages, while it was not reliably correlated with alpha lag coherence rhythm across cohorts and INT estimation methods. At the individual level, when averaging across electrodes, INT length was positively correlated with alpha lag coherence burst at 16 months but not reliably at 6, or 9 months. No relationship between INT and lag coherence rhythm was found to be significant in both the exploratory and validation cohorts for both INT estimation methods at the individual level.

## Discussion

Using longitudinal EEG data at 6, 9, and 16 months, we confirmed that INT shorten during infancy and remain longer in infants than in adults across conditions. Infant INT also correlated with properties of alpha-band activity. These findings extend previous reports that neonates integrate information over longer timescales than adults (Truzzi and Cusack 2023; Yates et al. 2022), with integration windows decreasing with age and experience, while overcoming limitations of previous studies.

### Development of intrinsic timescales

Here, we corroborated and extend previous neonatal findings (Truzzi and Cusack 2023) by showing that INT remain longer than in adults at least up to 16 months, generalizing previous fMRI results to EEG. Importantly, these effects were preserved after controlling for sociodemographic factors and trial sampling and are not attributable to confounds such as the slower infant blood-oxygen-level dependent (BOLD) response or sleep-state differences. The results also generalize across temporal scales, consistent with scale-invariance of INT in adults and other mammals. Recently, researchers suggested that scale-invariant properties like INT are at the root of coordination of activity across many scales and are phenomena occurring broadly in the natural world and relating to criticality, which indicates how close a system is to dynamic stability and is marked by scale and spatial invariant fluctuations (Cocchi et al. 2017). Wu and Gollo (2025) found that elderly adults have overall shorter intrinsic timescales compared to young adults and suggested that this is a marker of the elderly brain being further away from a critical functioning point, hence, more stable but efficient. From this perspective, intrinsic timescales may shorten during periods of increased functional specialization but then lengthen again during developmental windows characterized by increased plasticity, such as infancy and adolescence, when a more flexible and adaptive regime may be advantageous. Importantly, a recent preprint examining intrinsic timescales in youth reports that ITS increase again during adolescence. This finding is consistent with the possibility that developmental changes in INT are nonlinear, with timescales lengthening during periods of heightened plasticity, when increased flexibility in information processing may be beneficial (Shafiei et al. 2026). In line with this interpretation, our findings suggest that during infancy, the brain is on average in a more flexible and adaptable state, but not at the same level for all infants. However, we emphasize that these interpretations remain speculative, and establishing the full developmental trajectory of intrinsic timescales across the lifespan is an important direction for future research. Other measures have also been used in adults to characterize temporal dependencies in neural signals, including autocorrelation-based metrics such as the autocorrelation window (eg ACW-1 and ACW-0.5), as well as scale-free measures such as the Hurst exponent (Stanyard et al. 2024). These approaches index related aspects of temporal structure but are not fully equivalent, and their correspondence with intrinsic timescales, particularly in infancy, remains an open question.

Importantly, the infant INT spatial distribution differed from adults and could not be predicted from adult patterns, indicating that the hierarchical organization seen in adulthood is not yet present. We also found no robust region-specific trajectories, suggesting an early non-hierarchical organization, consistent with Yates et al. (2022). However, results also show that there is a large individual variability across infants. Together, these results demonstrate that both the duration and spatial organization of INT change from infancy to adulthood, implying that infants may parse sensory input differently from adults but that how the sensory information is parsed could be distinct across individual infants. This has important implications for developmental research, as experimental paradigms involving temporally extended stimuli may not be processed by infants in the same way as by adults and a same stimulus that changes in time could be processed differently by distinct infants.

### Alpha rhythm contributions to integration

Electrode-wise averages across individuals showed that INT were associated with alpha-band rhythmicity, but not reliably to alpha-band oscillatory properties. The relation to alpha-band rhythmicity supports the idea that INT arose from regional neural circuit dynamics rather than being the consequence of hemodynamic fluctuation properties or epiphenomena. The lack of consistent individual-level correlations indicates INT are not simply driven by differences in brain maturation, ie the average individual alpha peak frequency which increases with age (Freschl et al. 2019, 2021) but instead relate to differences in brain activity across areas. The only exception to this pattern was the positive correlation between INT and alpha, which was significant both in space and at the individual level. However, since this result appeared only at one time point, more research is necessary before its interpretation can be clarified.

As expected, alpha self-predictability at short ranges positively correlated with the distribution of INT values over the scalp, and this pattern also appeared at the individual level. Therefore, systematic alpha variations positively relate to longer periods of self-predictability. However, when examining alpha self-predictability at longer ranges, this relation disappears. Alpha oscillations are linked to adaptive executive control and to the ability to efficiently modulate attention (Murphy et al. 2014; Sadaghiani and Kleinschmidt 2016). Differences in alpha power and phase synchronization also underlie variations in stimulus processing in neurodivergent populations, such as individuals with ASD or attention-deficit/hyperactivity disorder (ADHD) (ter Huurne et al. 2013; Murphy et al. 2014). Given the association between alpha rhythm and INT and the proposed link between INT and criticality (Wu and Gollo 2025), it is possible that INT influences how individuals integrate and select information over time, modulating the developmental trajectory of their cognitive and social abilities. These mechanisms in the future may also have relevance for understanding brain function in clinical conditions such as autism, ADHD, epilepsy, or dementia. However, this remains a speculative hypothesis that was not tested in the present study and will require dedicated investigation in future research.

### Limitations and future directions

This study has several limitations that guide future work. Although our protocols were more comparable across infants and adults than in previous fMRI studies, baseline differences may remain (Camacho et al. 2020), and infants’ cognitive states may vary by age even under the same stimulation. Peripheral measures (eg ECG, eye-tracking; Xie et al. 2018) could better control for such differences and capture transient INT variations which were not considered in this project because, due to methodological constraints, we averaged across epochs assuming stationarity of brain activity. While outside the scope of this study, other frequency ranges (theta, beta, gamma) that are linked to working memory and consolidation (Lundqvist et al. 2016; Miller et al. 2018) may further delineate the correlates of INT. Moreover, INT measures may be influenced by aperiodic brain activity, which undergoes marked developmental change and are thought to represent more stable activity (Voytek and Knight 2015; Schaworonkow and Voytek 2021; Rico-Picó et al. 2023; Wilkinson et al. 2024). Future approaches that reduce oscillatory impact (eg Cusinato et al. 2023) may help isolate aperiodic contributions. Finally, while we hypothesize that INT support abstract learning, their direct relationship with cognition was not tested here. Linking INT to paradigms such as sequence learning could clarify their role in development.

### Conclusion

Research about INT in the infant brain is just beginning, and our results provide a much-needed foundational knowledge by shedding light on INT developmental trajectory, corroborating findings showing longer INT in young infants and a gradual shortening of INT values during early development, generalizing the findings from fMRI to EEG and suggesting that these effects occur independently of the arousal state and neuroimaging technique. In conclusion, we showed that longer INT in early infancy are a feature of the infant brain, and considering how the brain integrates information over time during the first year of life should be included in future research investigating learning and development. Understanding the neural mechanisms underlying these longer timescales, as well as their shortening over development, opens the path to future research studying how brain processing timescales relate to cognitive development and learning processes and whether they relate to neurodivergent developmental trajectories.

## Supplementary Material

its_eeg_CerebralCortex_supplementary_submission_final_bhag077

## Data Availability

Raw data are confidential because they contain sensitive information about infants. The anonymized data reporting the estimated intrinsic timescales together with the code used to process and analyze the data are published in a public GitHub repository (https://github.com/AnnaTruzzi/longitudinal_EEG_timescales).
