# Supplementary material for: The longitudinal development of intrinsic timescales in infancy and their relation to alpha brain rhythm
Source: Cereb Cortex. 2026 Jun 24;36(6):bhag077. doi: 10.1093/cercor/bhag077 (PMC13293257; doi:10.1093/cercor/bhag077)
Supplement: its_eeg_CerebralCortex_supplementary_submission_final_bhag077 [file its_eeg_cerebralcortex_supplementary_submission_final_bhag077.docx]

**The longitudinal development of intrinsic timescales in infancy and their relation to alpha brain rhythm**

Anna Truzzi^1^, Josué Rico-Picó^2^, Maria Rosario Rueda^3,4^, Rhodri Cusack^5,6^

1. School of Psychology, Queen’s University Belfast, UK

2. Department of Biobehavioural Sciences, Teachers College, Columbia University, New York, United States of America

3. Mind, Brain and Behaviour Research Center, University of Granada, Spain

4. Department of Experimental Psychology, University of Granada, Granada, Spain

5. School of Psychology, Trinity College Dublin, Dublin, Ireland

6. Trinity College Institute of Neuroscience, Trinity College Dublin, Dublin, Ireland

**Running title**: Longitudinal development of intrinsic timescales and their relation to alpha brain rhythm

**Corresponding author**

Anna Truzzi, Ph.D.

a.truzzi@qub.ac.uk

Room 03.534, David Keir Building, 18-30 Malone Road, Belfast, BT9 5BN, UK

**SI. Socioeconomic and maternal mental health of the sample**

As is characteristic of developmental studies with infants, some of the participants had missing data either because they did not attend the visit or because of technical difficulties in both cohorts, which might bias the results of the longitudinal analysis (Enders, 2013; Matta et al., 2018). Infants who attended the second and third visit (vs. those who did not) did not differ in socioeconomic status (SES), a composite of household income, parents’ education, and parents’ occupations, house organization, or maternal depression measured using CHAOS (Matheny et al., 1995) and BDI-II (Beck et al., 1996) questionnaires, respectively (all *p*s > .38). However, given that the data were missing completely at random (MCAR) in both cohorts (exploratory: Ⲭ = 5.27, *p* = .51; validation: Ⲭ = 2.29, *p* = .98) accordingly Little’s test results, we estimated the missing values in the analysis. We initially tested whether missing values in the validation and exploratory cohorts differed in three aspects of the home environment (socioeconomic status, house organization, and maternal mental health) using a Mann-Whitney test. Neither in the exploratory cohort (9-mo. - SES: U = 102.5, *p =* .94, CHAOS: U = 125, *p =* .83, BDI: U = 166, *p =* .27; 16-mo. - SES: U = 161.5, *p =* .78, CHAOS: U = 187, *p =* .57, BDI: U = 190, *p =* .63) nor in the validation cohort (9-mo. - SES: U = 77.5, *p =* .78, CHAOS: U = 136, *p =* .47, BDI: U = 101, *p =* .62; 16-mo. SES: U = 160, *p =* .58; CHAOS: U = 193.5, *p =* .91; BDI: U = 231, *p =* .38) there were differences between the participants who attended (vs. did not attend). Additionally, we tested whether the exploratory cohort and validation cohort groups differed in their environmental characteristics using the Mann-Whitney test between groups to check whether the pseudo randomization properly created equivalent groups. None of the analyses revealed significant differences between the SES (U = 661.5, *p* = .71), maternal depression (U = 692, *p* = .23), and home organization (U = 914, *p* = .38) groups. Indeed, the punctuations of both groups (Table S1) were similar on average when the component variables were considered.

All families who took part in the study had at least one parent from Spain except for 2 families (one of Arabic descent, one of Latin America descent). Families with multiple nationalities were as follows: one had mother of Spanish descent and father of USA descent, 1 had mother of Eastern Europe descent and father of Spanish descent, one had mother of Portuguese descent and father of Spanish descent. Most children were only exposed to Spanish at home (84%). Bilingual families (n = 11) mostly talked in English to their children (n = 7), but there were also parents who spoke Portuguese (n = 1), Euskera (n = 1), Arabic (n = 1), or German (n = 1). Of the participants who attended the first session, 36 and 40 mothers completed the Beck’s Depression Inventory (BDI)-II in the exploratory and validation cohorts, respectively. Most of the mothers (main: 72.09%, validation: 71.79%) scored had punctuations within the minimal depression range, while 13.25% and 15.38% had values within the moderate range, and the rest fell within the mild depression range.

In the income-to-need ratio (i.e., salary divided by federal poverty federal line for families with the same demographic composition by the Statistics National Institute), we found that 18 and 16 families had an income below the poverty line for the exploratory and validation cohorts, respectively. Most of the remaining families had an income between one and two times the poverty income (exploratory N = 26, validation N = 33), while the rest earned twice or more (exploratory N = 14, validation N = 15). With respect to education, mothers presented at least a basic school degree (exploratory N = 1, validation N = 2), and the majority presented either a bachelor's degree (exploratory N = 9, validation N = 7) or postgraduate studies (exploratory N = 12, validation N = 10). Among the remaining mothers, 15 had achieved a superior high school diploma (exploratory N = 9), 10 had a middle school diploma (exploratory N = 2), and 5 had completed compulsory schooling (exploratory N = 3). In the case of fathers, only one in the exploratory cohort did not finish basic schooling, while five (exploratory N = 2) and 18 (exploratory N = 7) had completed compulsory and middle school diplomas, respectively. In addition, 14 fathers (seven per group) had a postgraduate degree, and another 14 fathers (exploratory N = 9) had a bachelor's degree. The rest had either a medium technical degree (N = 9, exploratory N = 7) or a superior technical degree (N = 5, exploratory N = 5). According to the National Classification of Occupation (2011), the largest percentage of mothers were either unemployed (N = 35, exploratory N = 17) or presented highly qualified jobs related to medicine, management, teaching, or science (N = 24, exploratory N = 10). Additionally, 19 (exploratory N = 12) and 14 (exploratory N = 5) mothers worked as technicians or in office positions, respectively. The rest worked either in non-qualified jobs (N = 7, exploratory N = 4), bars, and restaurants (N = 3, exploratory N = 1), or as caregivers (N = 2, exploratory N = 1). Similarly, most fathers worked in fields related to engineering, medicine, teaching, or science (N = 39, exploratory N = 16), and only one in the exploratory cohort related to management. In addition, a large percentage of fathers worked in office positions (N = 31, exploratory N = 17), and 10 were unemployed (exploratory N = 8). Of the rest, five fathers had non-qualified jobs (exploratory N = 1), nine fathers had basic jobs related to mechanics and agriculture (exploratory N = 5), and 18 worked in bars and restaurants or in caregiving jobs (exploratory N = 7).

**Table S1.**

*Maternal depression, socioeconomic status, and CHAOS (mean, standard deviation) for the participants included in the exploratory and validation cohorts in the analysis.*

| **Cohort** | **BDI** | **SES** | **CHAOS** |
| --- | --- | --- | --- |
| **Exploratory** | 10.05 (7.55) | 0.2 (0.85) | 43.7 (13.98) |
| **Validation** | 11.69 (7.59) | 0.09 (0.77) | 40.56 (13.6) |


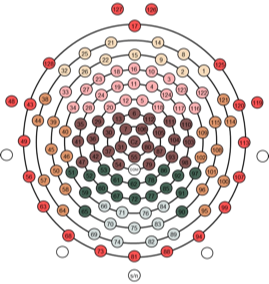
**Fig. S1. EEG net layout employed in the study.** The colours indicate the cluster of each electrode: red (removed), light green (occipital), green (parietal), light brown (temporal), brown (central), pink (frontal), and beige (frontal pole).

**SII. Intrinsic timescales: model fit and missing values**

The autocorrelation function (ACF) of EEG signal occasionally resembles a dampened sinusoid (Tang et al., 2025) possibly due to the rhythmicity of the signal, however, since the τ coefficient is estimated on the fitted exponential curve, an eventual delayed raise in autocorrelation values is not affecting the estimation of the intrinsic timescales for each channel (See Fig S2 for examples of how the exponential curve fits the autocorrelation function when ITS is short vs long). To evaluate the fit of our exponential curve to the ACF we calculated the R^2^ for each model and then average across participants for each age. The R denoted a good fit for all ages (*Exploratory cohort* - 6mo: R^2^ = 0.69; 9mo: R^2^ = 0.70; 16mo: R^2^ = 0.73; *Validation cohort* - 6mo: R^2^ = 0.68; 9mo: R^2^ = 0.72; 16mo: R= 0.71; *Adults* - R^2^ = 0.68. When the model used to quantify ITS (τ values) failed to converge – due to noisy ACF or to the constraints we set on the model parameters - the ITS of an electrode could not be computed. Therefore, we also excluded epochs with more than 25% missing values after the tau calculation. The number of retained participants and epochs compared with the original numbers is shown in Table S2. further details about the missing values such as their percentage in the different clusters of electrodes are found in Tables S4-5. Additionally, a visualisation of the distribution of missing values in the infant and adult datasets is shown in Figure S3. For the exploratory group, at 6 months, the model failed to converge in 4.51% of the cases, and in any single electrode, never more than 11% of the cases. At nine months, the model failed to converge in 5.44% of cases and never more than 11% of cases for each electrode. At 16 months, the model failed to converge in 4.76% of the cases and never more than 11.67% of the cases for each electrode. The validation group had a very similar pattern with an average of 4.95%, 4.79%, and 4.39% cases, with never more than 9.2%, 10.26%, and 9.81% of cases per electrode at 6, 9, and 16 months, respectively. In adults, the model failed to converge in 5.03% of the cases, and in any single electrode, never more than 10.03% of the cases. Epochs with more than 25% of non-convergent electrodes were removed from further processing. Otherwise, the tau value of the electrodes that did not converge were computed by averaging the τ values of the proximal electrodes within the cluster and epoch. For visualization of the distribution of missing values across ROIs in the three groups, see Fig. S4-5 in the Supplementary Information. The percentage of non-convergent electrodes did not vary between visits (Exploratory Cohort B _Age_= 0.01, *p* = .279, Validation Cohort B _Age_= -0.002, *p* = .829).


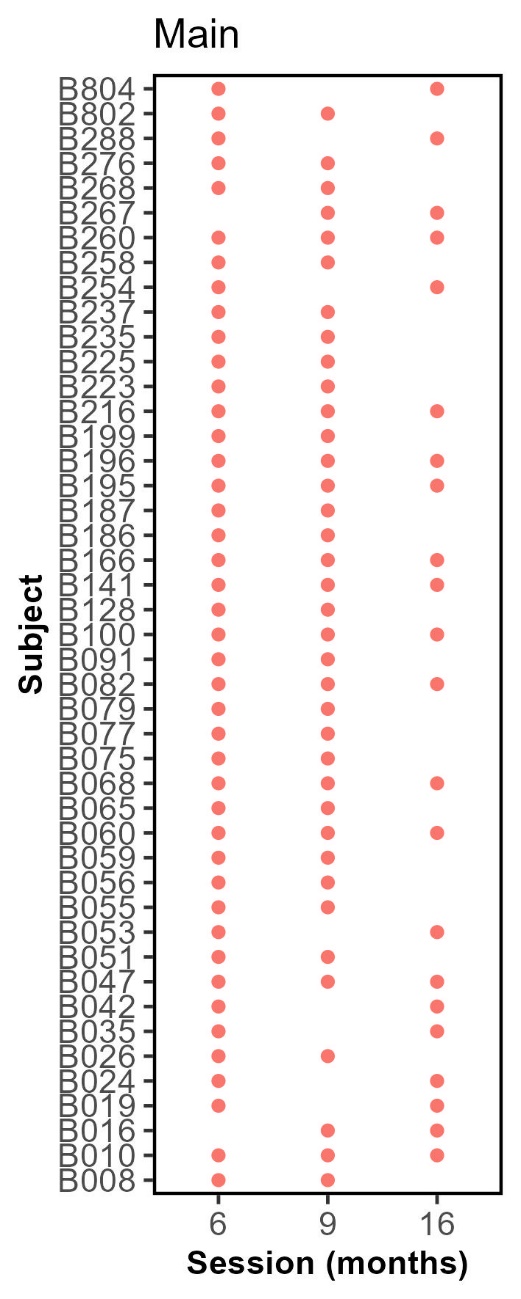

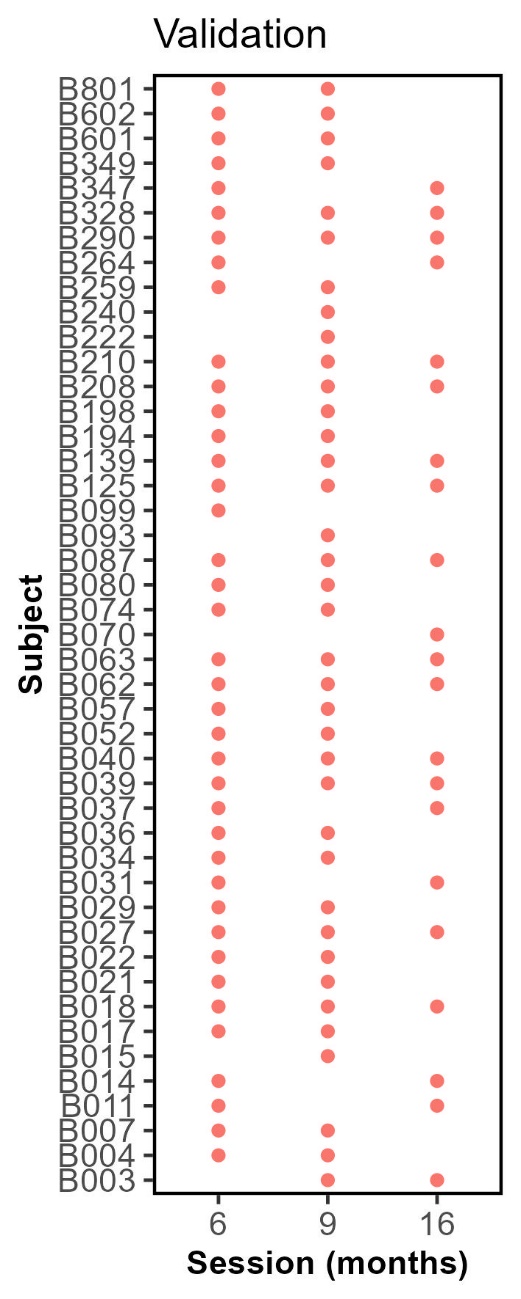


**Fig. S2. Missing data distribution in the main and validation longitudinal cohorts.** The figure displays whether a participant attended to the session (dot) or did not (empty space) for each one of the sessions.


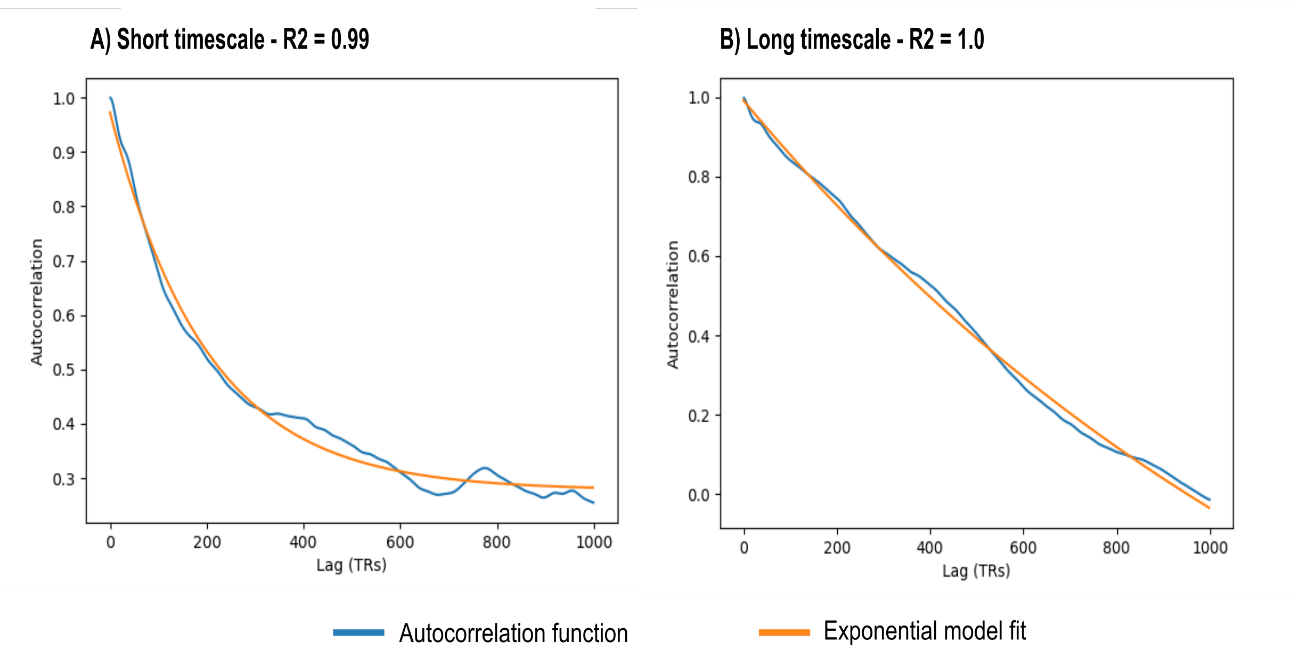


**Figure S3.** Modelling the autocorrelation function (ACF) with an exponential function. This figure shows how the ACF (in blue) of a given participant, channel, and epoch, was fitted by an exponential function (in orange) in the case of (A) a fast decay ACF (short timescale), or (B) a slow decay ACF (long timescale). The R2 in the title expresses how well the exponential modelled the ACF.

**Table S2.**

*Number of participants and epochs before and after removing epochs with high levels of motion and epochs where the model failed to estimate the intrinsic timescales for more than 25% of electrodes.*

|  | **Exploratory Cohort** | | | **Validation Cohort** | | |
| --- | --- | --- | --- | --- | --- | --- |
|  | *Original sample* | *Low movement only* | *<25% nan only* | *Original sample* | *Low movement only* | *<25% nan only* |
| **6-mo.** | N=45  Epochs=449 | N=43  Epochs=191 | N=43  Epochs=186 | N=45  Epochs=472 | N=40  Epochs=174 | N=39  Epochs=164 |
| **9-mo.** | N=37  Epochs=359 | N=37  Epochs=282 | N=37  Epochs=272 | N=37  Epochs=361 | N=37  Epochs=312 | N=37  Epochs=304 |
| **16-mo.** | N=22  Epochs=216 | N=22  Epochs=180 | N=22  Epochs=170 | N=23  Epochs=236 | N=22  Epochs=173 | N=21  Epochs=164 |
| **Adults** | N=11  Epochs=339 | N=11  Epochs=339 | N=11  Epochs=324 | - | - | - |

**Table S3.**

*Percentage (mean, standard deviation) of non-convergent autoregressive models computing the intrinsic time scale values per cohort, session, and sex. The table excluded bad trials and those epochs with a percentage of nan values superior to 25%.*

| **Cohort** | **Session** | **Sex** | **Cluster** | | | | | |
| --- | --- | --- | --- | --- | --- | --- | --- | --- |
|  |  |  | *C* | *Fr* | *Fp* | *O* | *P* | *T* |
| **Exploratory**  **Cohort** | *6-mo.* | F | 0.06 (0.24) | 0.05 (0.22) | 0.05 (0.21) | 0.04 (0.2) | 0.05 (0.22) | 0.04 (0.19) |
|  |  | M | 0.05 (0.21) | 0.03 (0.17) | 0.04 (0.2) | 0.03 (0.17) | 0.03 (0.18) | 0.03 (0.17) |
|  | *9-mo.* | F | 0.06 (0.23) | 0.04 (0.2) | 0.04 (0.2) | 0.02 (0.15) | 0.04 (0.19) | 0.05 (0.21) |
|  |  | M | 0.05 (0.22) | 0.05 (0.22) | 0.04 (0.2) | 0.02 (0.15) | 0.04 (0.2) | 0.04 (0.19) |
|  | *16-mo.* | F | 0.04 (0.21) | 0.04 (0.2) | 0.04 (0.2) | 0.07 (0.25) | 0.05 (0.22) | 0.04 (0.2) |
|  |  | M | 0.04 (0.19) | 0.03 (0.18) | 0.04 (0.2) | 0.04 (0.2) | 0.04 (0.21) | 0.03 (0.17) |
| **Validation**  **Cohort** | *6-mo.* | F | 0.04 (0.2) | 0.03 (0.18) | 0.04 (0.2) | 0.04 (0.2) | 0.02 (0.16) | 0.02 (0.13) |
|  |  | M | 0.05 (0.21) | 0.04 (0.19) | 0.06 (0.23) | 0.03 (0.17) | 0.03 (0.16) | 0.04 (0.19) |
|  | *9-mo.* | F | 0.05 (0.21) | 0.04 (0.2) | 0.04 (0.19) | 0.04 (0.19) | 0.04 (0.19) | 0.05 (0.21) |
|  |  | M | 0.06 (0.23) | 0.04 (0.19) | 0.03 (0.18) | 0.03 (0.18) | 0.04 (0.2) | 0.04 (0.2) |
|  | *16-mo.* | F | 0.04 (0.2) | 0.03 (0.16) | 0.04 (0.2) | 0.03 (0.17) | 0.03 (0.18) | 0.03 (0.18) |
|  |  | M | 0.03 (0.17) | 0.03 (0.16) | 0.03 (0.18) | 0.09 (0.29) | 0.04 (0.2) | 0.02 (0.15) |

**Note.** F, female; M, male; C, central; Fr, frontal; Fp, frontal pole; O, occipital; P, parietal; T, temporal.

**Table S4.**

*Percentage (mean, standard deviation) of non-convergent autoregressive models computing the intrinsic time scale values in the adult cohort. The table excluded bad trials and those epochs with a percentage of nan values superior to 25%.*

| **Condition** | **Cluster** | | | | | |
| --- | --- | --- | --- | --- | --- | --- |
|  | *C* | *Fr* | *Fp* | *O* | *P* | *T* |
| **Video** | 0.05 (0.23) | 0.05 (0.22) | 0.05 (0.21) | 0.03 (0.16) | 0.03 (0.16) | 0.06 (0.24) |
| **Eyes**  **Closed** | 0.03 (0.17) | 0.03 (0.17) | 0.03 (0.17) | 0.02 (0.14) | 0.02 (0.14) | 0.04 (0.18) |
| **Eyes**  **Open** | 0.06 (0.23) | 0.08 (0.27) | 0.1 (0.3) | 0.04 (0.19) | 0.03 (0.18) | 0.05 (0.22) |

**Note.** C, central; Fr, frontal; Fp, frontal pole; O, occipital; P, parietal; T, temporal.


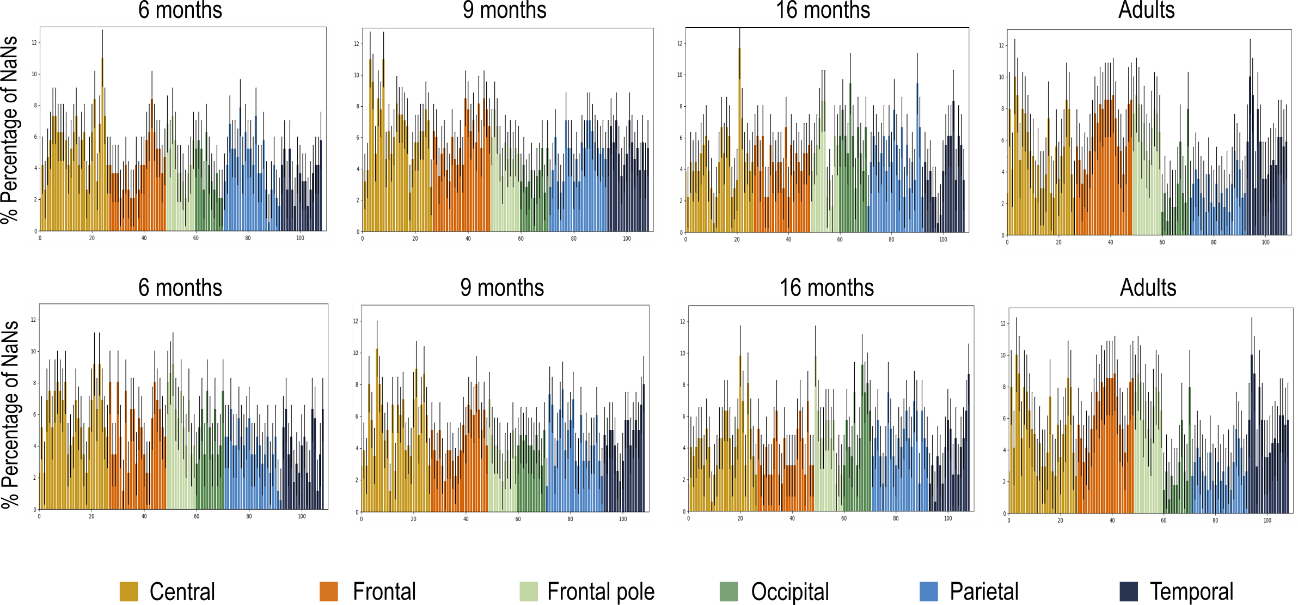


**Fig. S4.** Percentage of nan values for each electrode in the different infant sessions and in the adult sample. The different colours represent different clusters as detailed in the legend. The adults’ plot is replicated twice for ease of comparison with the exploratory and validation cohorts. A) Exploratory cohort. B) Validation cohort.


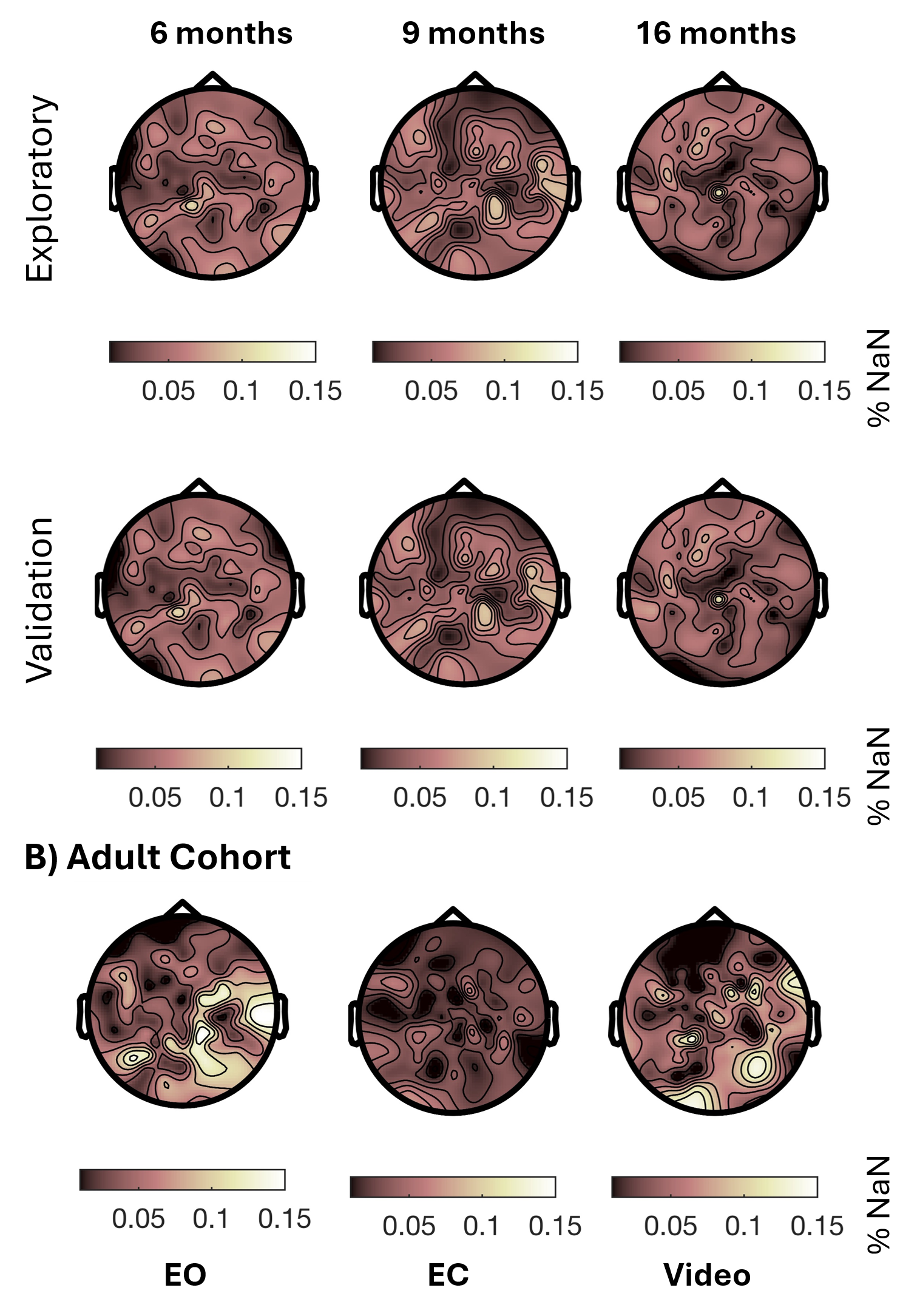


**Fig. S5.** Topographical representation of the percentage of non-convergent intrinsic timescales models (NA values) per age and condition in all the cohorts.


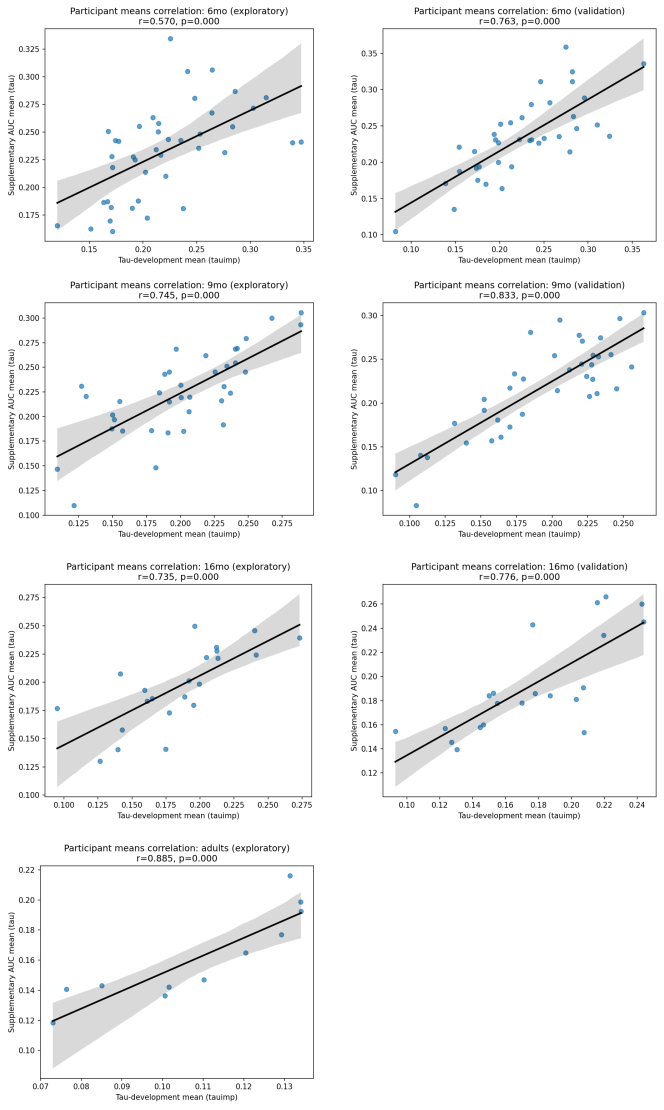


**Figure S6. Correlation between intrinsic timescales estimated from the exponential curve (INT) and using the area under the curve method (INT_AUC).**


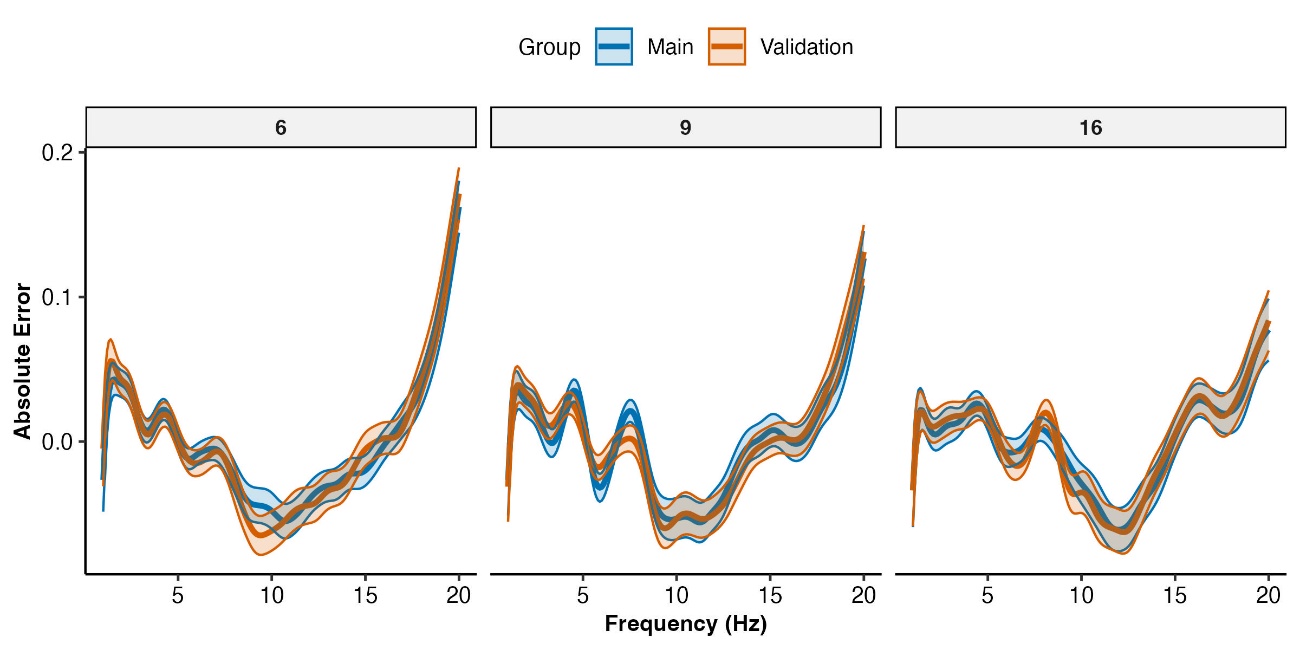

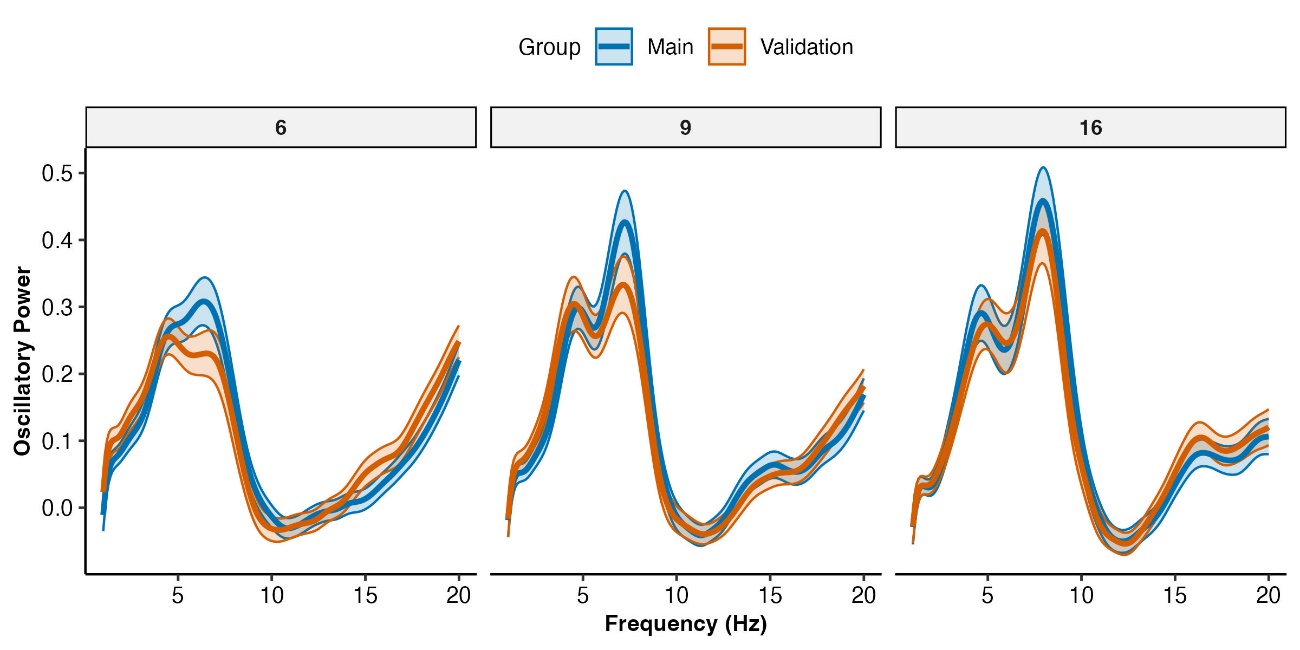
**Figure S7. Absolute error and oscillatory power spectrum divided by age and cohort.** The graph shows the mean oscillatory power spectrum and absolute error (log10(mV^2/Hz)) per cohort and age averaged across clusters. Shaded area represent the standard error.

**SIII. Longitudinal development of tau value: Robustness of the longitudinal effects**

To determine the robustness of developmental we reran the final models of the main test -random slope of time per participant, plus time, time squared, and area as fixed effects- including other variables of interest that has been previously related to electrophysiological brain function as covariates: SES, maternal mental health, gestational information. Socioeconomic status consisted of the parameter mentioned in SI (Income to Needs, Maternal and Paternal education, and Maternal and Paternal occupation) while the BDI was used as a proxy of maternal mental health. Given the differences in scale between tau values and covariates, all covariates were standardized before being included in the model. Missing values at 6 months were replaced, if possible, by the same information provided by the families at 16 months. In case both 6- and 16-months old questionaries were missing, the mean across participants was considered to retain the same number of participants. The results after introducing the covariates were consistent with the main test in both cohorts. In the Exploratory cohort, the tau values got reduced across sessions (B = -.10, *p* < .001) with a quadratic positive effect (B = .09, *p* < .001), which also occurred in the validation sample (B _time_ = -.18, *p* < .001; B _time-squared_ = .17, *p* < .001).

In a second step, we aimed to determine if the results found could have been driven by the difference in the trials between sessions. To address data amount difference, and test whether the data selection could explain the results, we reran the models selecting the same number of trials per participant – 3 trials (i.e., 30s of data). This procedure was bootstrapped (iterations = 5000 with replacement) in which the final model was computed with and without the sociodemographic and environmental variables. Then we computed the average estimates and confidence intervals of time and time squared fixed effects and constructed the standard error and confidence intervals. As can be seen in Fig. S6 the beta estimates were consistent with the results found with all the trials included in the models and never crossed zero accordingly the confidence intervals (see also Table S5).

**Table S5**

*Beta estimates of the different models testing the development of intrinsic timescales in exploratory and validation cohorts. The table provides information about the Beta estimate, (standard error), and [95% Confidence Interval] for models with all the trials (No Boot), bootstrapping the trials (Boot.) while including EEG covariates (Basic) or EEG covariates plus sociodemographic variates (Complete).*

| **Cohort** | **Fixed Effect** | **w/o Sociodemographic Covs.** | | **w/ Sociodemographic Covs.** | |
| --- | --- | --- | --- | --- | --- |
|  |  | *Boot* | *No Boot.* | *Boot* | *No Boot.* |
| **Exploratory** | *Time* | -0.09 (0.02) [-0.11, -0.04] | -0.12 (0.03) [-0.16, -0.08] | -0.09 (0.02) [-0.12, -0.05] | -0.12 (0.03)  [-0.16, -0.08] |
|  | *Time Squared* | 0.09 (0.03)  [0.02, 0.1] | 0.09 (0.03) [0.04, 0.14] | 0.09 (0.02) [0.03, 0.1] | 0.09 (0.03) [0.05, 0.14] |
|  | *Intercept* | 0.17 (0.01)  [0.16, 0.18] | 0.16 (0.01) [0.15, 0.18] | 0.18 (0.01) [0.16, 0.19] | 0.17 (0.01) [0.16, 0.18] |
| **Validation** | *Time* | -0.18 (0.02) [-0.22, -0.15] | -0.19 (0.03) [-0.23, -0.15] | -0.18 (0.02) [-0.22, -0.14] | -0.19 (0.03) [-0.23, -0.15] |
|  | *Time Squared* | 0.17 (0.03)  [0.13, 0.22] | 0.18 (0.03) [0.13, 0.23] | 0.16 (0.03) [0.12, 0.21] | 0.17 (0.03) [0.12, 0.22] |
|  | *Intercept* | 0.16 (0.01)  [0.15, 0.18] | 0.16 (0.01) [0.15, 0.17] | 0.18 (0.01) [0.17, 0.19] | 0.17 (0.01) [0.16, 0.18] |

Note. Bootstrapped CI for the non-trial bootstrapped approach was computed in 5000 iterations.


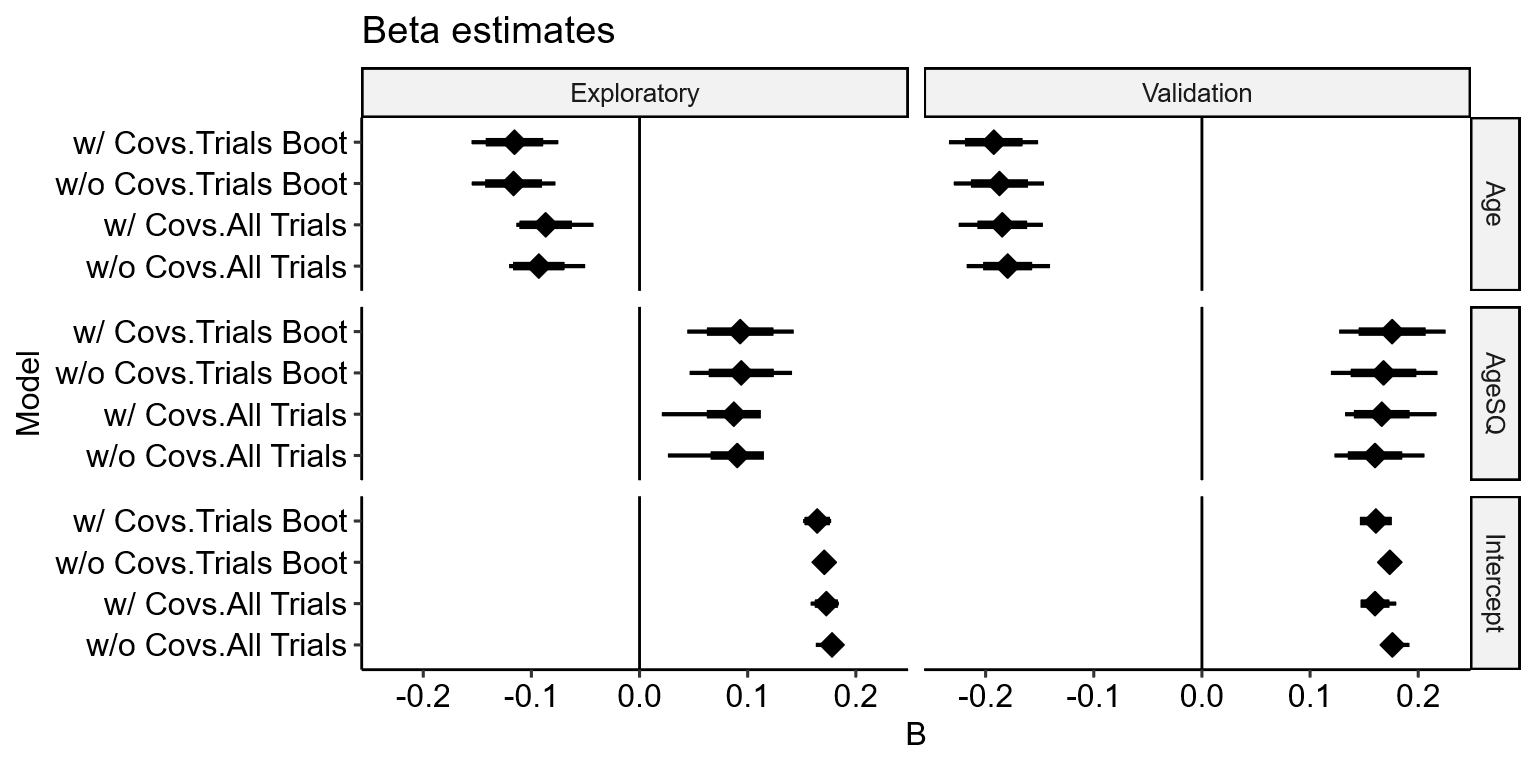


**Fig. S8. Standardized beta estimates for the linear mixed models exploring the development of intrinsic times scales.** The figure displays the mean beta, its standard error and 95% CI for time, time squared and intercept fixed effects in the Exploratory and Validation cohorts. The models displayed was either constructed with all the trials or with the same number of trials per participant (Trials Boot.). Additionally, these were constructed included percentage of nonconvergent electrodes (w/o Covs.) or included other sociodemographic variables of interest (w/ Covs.). CI in the regular models was extracted from 5000 bootstrapped iterations.

**Table S6.**

*Tau values (mean, standard deviation) were divided by session, sex, and cluster in the exploratory cohort.*

| **Session** | **Sex** | **Cluster** | | | | | |
| --- | --- | --- | --- | --- | --- | --- | --- |
|  |  | *Central* | *Frontal* | *Frontalpole* | *Occipital* | *Parietal* | *Temporal* |
| **6-mo.** | *F* | 0.22 (0.11) | 0.23 (0.12) | 0.23  (0.10) | 0.24 (0.12) | 0.22 (0.11) | 0.22 (0.11) |
|  | *M* | 0.21 (0.13) | 0.20  (0.11) | 0.21  (0.11) | 0.21 (0.1) | 0.22 (0.12) | 0.22 (0.13) |
| **9-mo.** | *F* | 0.19 (0.09) | 0.19 (0.09) | 0.22  (0.09) | 0.2 (0.07) | 0.20  (0.1) | 0.19 (0.08) |
|  | *M* | 0.2  (0.08) | 0.22 (0.09) | 0.21  (0.07) | 0.21 (0.07) | 0.20 (0.08) | 0.20 (0.08) |
| **16-mo.** | *F* | 0.18  (0.10) | 0.19 (0.08) | 0.20  (0.09) | 0.22 (0.09) | 0.18 (0.08) | 0.17 (0.08) |
|  | *M* | 0.17 (0.07) | 0.18 (0.06) | 0.18 (0.05) | 0.21 (0.06) | 0.19 (0.06) | 0.17 (0.06) |

**Table S7.**

*Tau values (mean, standard deviation) were divided by session, sex, and cluster in the validation cohort.*

| **Session** | **Sex** | **Cluster** | | | | | |
| --- | --- | --- | --- | --- | --- | --- | --- |
|  |  | *Central* | *Frontal* | *Frontalpole* | *Occipital* | *Parietal* | *Temporal* |
| **6-mo.** | *F* | 0.21 (0.13) | 0.22 (0.13) | 0.22 (0.13) | 0.24 (0.15) | 0.21 (0.12) | 0.19 (0.11) |
|  | *M* | 0.25 (0.14) | 0.23 (0.13) | 0.25 (0.14) | 0.27 (0.13) | 0.24 (0.13) | 0.25 (0.14) |
| **9-mo.** | *F* | 0.17 (0.07) | 0.18 (0.08) | 0.19 (0.08) | 0.2 (0.07) | 0.19 (0.08) | 0.19 (0.07) |
|  | *M* | 0.20 (0.09) | 0.19 (0.07) | 0.20 (0.08) | 0.21 (0.08) | 0.20 (0.08) | 0.20 (0.07) |
| **16-mo.** | *F* | 0.16 (0.07) | 0.17 (0.07) | 0.19 (0.07) | 0.19 (0.07) | 0.17 (0.08) | 0.16 (0.07) |
|  | *M* | 0.17 (0.06) | 0.17 (0.06) | 0.2 (0.07) | 0.22 (0.07) | 0.19 (0.08) | 0.19 (0.08) |


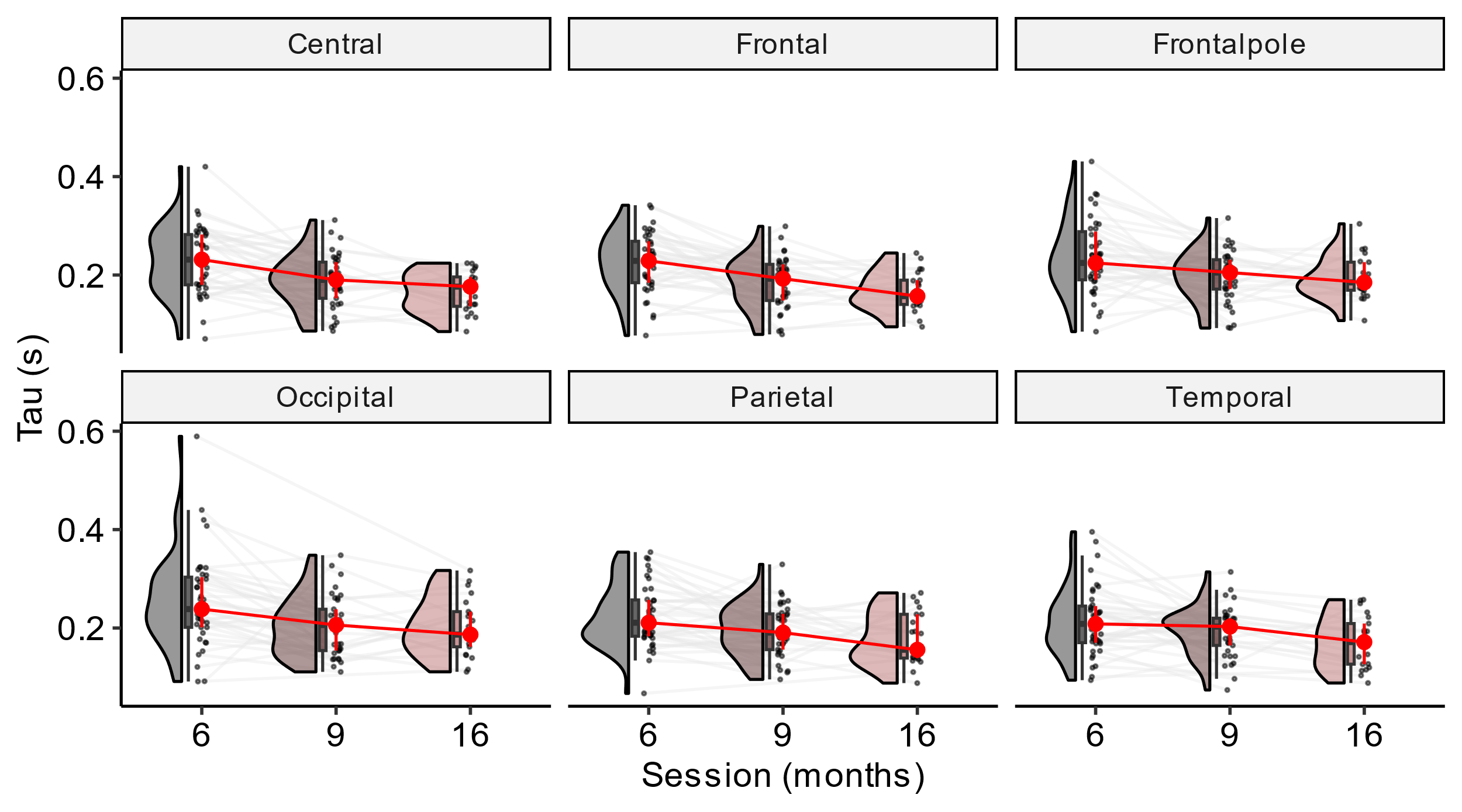

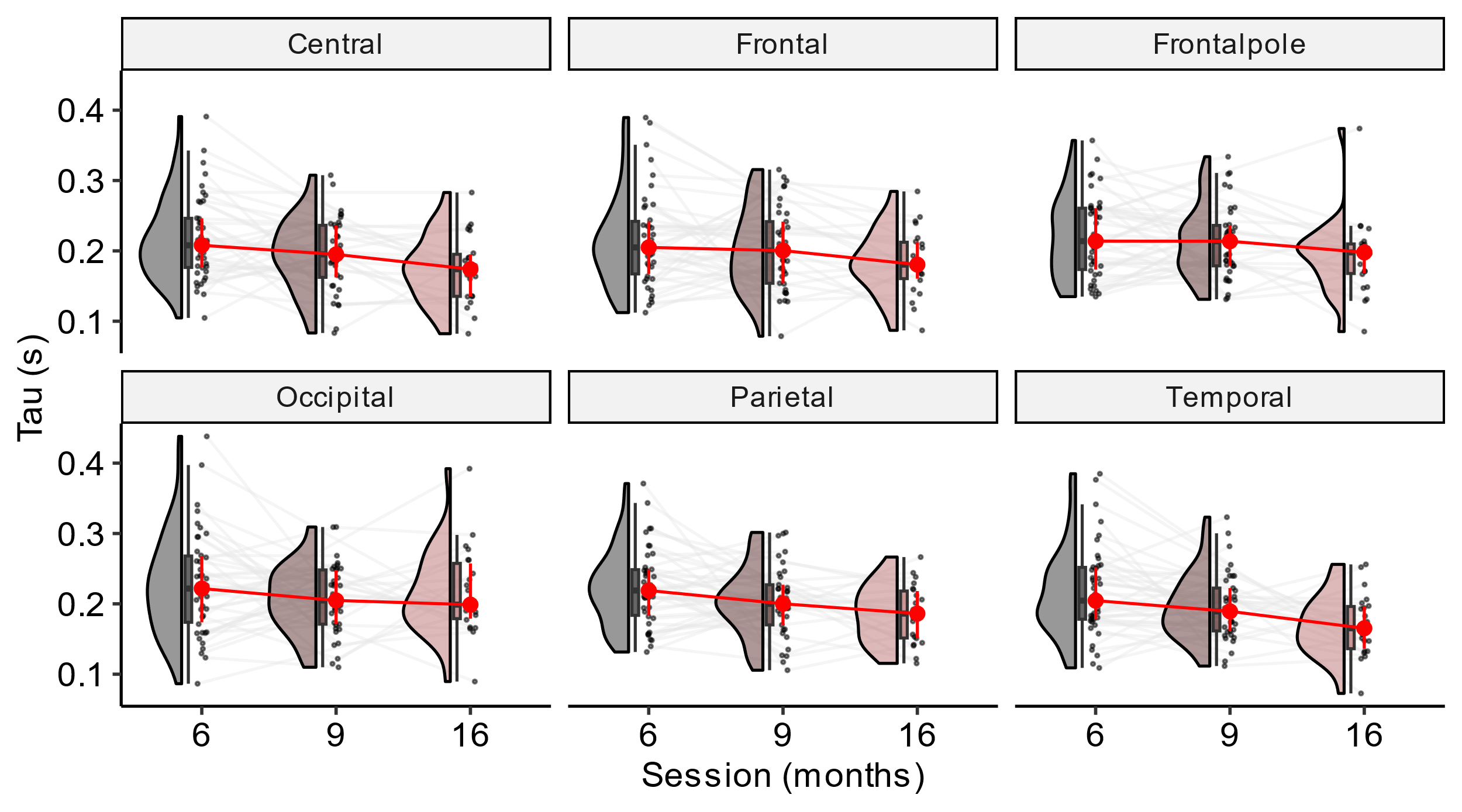


**A) Exploratory**

**B) Validation**

**Fig. S9. Intrinsic timescales development in the exploratory (A) and validation (B) cohorts per cluster.** Each dot corresponds to a participant, while the gray lines represent individual trajectories. Red dots and lines represent the median values and trajectory.

**Table S8.**

*Comparison between INT_AUC values’ distributions estimated with the area under the curve method in infants and adults for the different visits and conditions. Mann Whitney U statistics and Cohen’s d effect size are reported for each comparison. The INT_AUC values’ distributions comparisons were run separately for the infant exploratory and validation cohorts.*

| **Infant visit** | **Adult condition** | **Exploratory** | | **Validation** | |
| --- | --- | --- | --- | --- | --- |
|  |  | Mann Whitney U | *d* | Mann Whitney U | *d* |
| **6-mo.** | Video | 11841*** | 3.7 | 11816*** | 3.4 |
|  | EO | 11881*** | 4.8 | 11880*** | 4.4 |
|  | EC | 11661*** | 3.0 | 11532*** | 2.7 |
| **9-mo.** | Video | 11785*** | 3.3 | 11555*** | 2.6 |
|  | EO | 11881*** | 4.4 | 11856*** | 3.8 |
|  | EC | 11404*** | 2.6 | 10884*** | 2.0 |
| **16-mo.** | Video | 9540*** | 1.2 | 8882*** | 1.0 |
|  | EO | 11368*** | 2.4 | 11160*** | 2.1 |
|  | EC | 8697*** | 0.9 | 8319*** | 0.8 |

*. *p<.008, **p<.01, ***p<.001. EO = Eyes Open, EC = Eyes closed*

**Table S9**

*Mean and standard deviation of the betas distributions for the linear models predicting infant INT_AUC spatial distribution estimated with the area under the curve method starting from the average adult INT_AUC in different conditions. Confidence interval (CI) and t values resulting from testing whether the distributions are significantly different from zero are reported.*

| **Infant Visit** | **Adult Condition** | **Exploratory Cohort** | | | | **Validation Cohort** | | | |
| --- | --- | --- | --- | --- | --- | --- | --- | --- | --- |
|  |  | *M(SD)* | *CI* | *t* | *R^2^* | *M(SD)* | *CI* | *t* | *R^2^* |
| **6-mo.** | *Video* | 0.17  (0.79) | [-0.07, 0.42] | 1.4 | - | 0.50 (0.82) | [0.24, 0.78] | 3.8 | 0.03 |
|  | *EO* | 0.49 (0.85) | [0.23, 0.76] | 3.8 | 0.03 | 0.91  (0.82) | [0.64, 1.17] | 6.8 | 0.06 |
|  | *EC* | 0.28 (0.67) | [0.08, 0.49] | 2.8 | 0.04 | 0.57  (0.70) | [0.35, 0.80] | 5.1 | 0.07 |
| **9-mo.** | *Video* | 0.20  (0.62) | [-0.004, 0.41] | 2.0 | - | 0.07 (0.58) | [-0.13, 0.26] | 0.7 | - |
|  | *EO* | 0.45 (0.57) | [0.26, 0.65] | 4.8* | 0.04 | 0.41  (0.57) | [0.23, 0.60] | 4.4 | 0.04 |
|  | *EC* | 0.30  (0.56) | [0.11,  0.49] | 3.2 | 0.05 | 0.19 (0.42) | [ 0.05, 0.33] | 2.7 | 0.04 |
| **16-mo.** | *Video* | 0.34  (0.57) | [0.09,  0.60] | 2.3 | 0.04 | 0.30  (0.52) | [0.07, 0.53] | 2.7 | 0.03 |
|  | *EO* | 0.49  (0.65) | [0.19,  0.79] | 3.4 | 0.06 | 0.64  (0.63) | [0.35, 0.92] | 4.7 | 0.07 |
|  | *EC* | 0.30  (0.42) | [0.11, 0.50] | 3.3 | 0.06 | 0.37  (0.54) | [0.13, 0.62] | 3.2 | 0.07 |

**p<.008, **p<.01, ***p<.001.* EO = Eyes open, EC = Eyes closed.

**Table S12.**

*Power parameters in the correlation analysis between the INT and oscillatory power and peak frequency in the exploratory cohort.*

| **Parameter** | **Session** | **Sex** | **C** | **Fr** | **Fp** | **O** | **P** | **T** |
| --- | --- | --- | --- | --- | --- | --- | --- | --- |
| **Oscillatory**  **Power** | *6-mo.* | F | 0.24 (0.31) | 0.6 (0.46) | 0.63 (0.43) | 0.16 (0.28) | 0.19 (0.23) | 0.4 (0.44) |
|  |  | M | 0.39 (0.35) | 0.73 (0.57) | 0.86 (0.69) | 0.26 (0.39) | 0.33 (0.38) | 0.49 (0.4) |
|  | *9-mo.* | F | 0.27 (0.34) | 0.63 (0.42) | 0.61 (0.48) | 0.04 (0.35) | 0.18 (0.27) | 0.39 (0.39) |
|  |  | M | 0.17 (0.26) | 0.44 (0.36) | 0.42 (0.44) | -0.02 (0.18) | 0.06 (0.2) | 0.26 (0.27) |
|  | *16-mo.* | F | 0.12 (0.37) | 0.46 (0.43) | 0.46 (0.46) | -0.03 (0.18) | 0.01 (0.26) | 0.19 (0.42) |
|  |  | M | 0.39 (0.36) | 0.71 (0.37) | 0.66 (0.36) | 0.14 (0.29) | 0.34 (0.32) | 0.46 (0.3) |
| **Peak**  **Frequency** | *6-mo.* | F | 6.97 (0.59) | 6.81 (0.75) | 6.79 (0.72) | 6.74 (0.77) | 6.85 (0.6) | 6.56 (0.79) |
|  |  | M | 7.07 (0.56) | 6.62 (0.77) | 6.51 (0.77) | 7.06 (0.68) | 7.13 (0.74) | 6.6 (0.92) |
|  | *9-mo.* | F | 7.38 (0.4) | 7.03 (0.5) | 6.98 (0.44) | 7.3 (0.51) | 7.24 (0.41) | 7.08 (0.4) |
|  |  | M | 7.46 (0.58) | 7.16 (0.7) | 7.15 (0.63) | 7.6 (0.48) | 7.43 (0.48) | 7.12 (0.84) |
|  | *16-mo.* | F | 8.02 (0.52) | 7.84 (0.67) | 7.83 (0.65) | 7.75 (0.59) | 7.96 (0.48) | 7.79 (0.56) |
|  |  | M | 8.17 (0.47) | 8.04 (0.7) | 7.96 (0.83) | 8.16 (0.53) | 8.15 (0.41) | 7.93 (0.61) |
| **R^2^** | *6-mo.* | F | 1 (0) | 0.99 (0.01) | 0.99 (0.01) | 1 (0) | 0.99 (0) | 0.99 (0.01) |
|  |  | M | 0.99 (0) | 0.99 (0.01) | 0.98 (0.01) | 1 (0) | 0.99 (0) | 0.99 (0.01) |
|  | *9-mo.* | F | 1 (0) | 0.99 (0.01) | 0.99 (0.01) | 1 (0) | 0.99 (0) | 0.99 (0.01) |
|  |  | M | 0.99 (0) | 0.99 (0.01) | 0.99 (0.01) | 1 (0) | 0.99 (0) | 0.99 (0.01) |
|  | *16-mo.* | F | 1 (0) | 0.99 (0) | 0.99 (0) | 1 (0) | 1 (0) | 0.99 (0) |
|  |  | M | 1 (0) | 0.99 (0) | 0.99 (0.01) | 1 (0) | 1 (0) | 0.99 (0) |

**Note.** F, female; M, male; C, central; Fr, frontal; Fp, frontal pole; O, occipital; P, parietal; T, temporal.

**Table S13.**

*Power parameters in the correlation analysis between the INT and oscillatory power and peak frequency in the validation cohort.*

| **Parameter** | **Session** | **Sex** | **C** | **Fr** | **Fp** | **O** | **P** | **T** |
| --- | --- | --- | --- | --- | --- | --- | --- | --- |
| **Oscillatory**  **Power** | *6-mo.* | F | 0.35 (0.53) | 0.65 (0.65) | 0.59 (0.64) | 0.16 (0.31) | 0.26 (0.35) | 0.42 (0.48) |
|  |  | M | 0.44 (0.5) | 0.82 (0.65) | 0.75 (0.59) | 0.28 (0.36) | 0.38 (0.39) | 0.75 (0.59) |
|  | *9-mo.* | F | 0.32 (0.38) | 0.57 (0.51) | 0.63 (0.59) | 0.17 (0.42) | 0.27 (0.39) | 0.39 (0.42) |
|  |  | M | 0.51 (0.47) | 0.96 (0.7) | 0.9 (0.66) | 0.26 (0.4) | 0.41 (0.52) | 0.65 (0.43) |
|  | *16-mo.* | F | 0.08 (0.26) | 0.34 (0.44) | 0.19 (0.37) | 0.01 (0.5) | -0.02 (0.25) | 0.2 (0.33) |
|  |  | M | 0.28 (0.3) | 0.72 (0.47) | 0.58 (0.39) | -0.12 (0.36) | 0.15 (0.31) | 0.47 (0.36) |
| **Peak**  **Frequency** | *6-mo.* | F | 6.77 (0.53) | 6.58 (0.62) | 6.47 (0.68) | 6.94 (0.77) | 6.68 (0.58) | 6.35 (0.62) |
|  |  | M | 6.9 (0.64) | 6.64 (1.03) | 6.43 (0.86) | 6.55 (0.77) | 6.69 (0.55) | 6.29 (0.61) |
|  | *9-mo.* | F | 7.34 (0.5) | 7.02 (0.6) | 7.02 (0.52) | 7.12 (0.6) | 7.19 (0.61) | 6.8 (0.61) |
|  |  | M | 7.24 (0.56) | 7.01 (0.76) | 6.91 (0.66) | 7.24 (0.73) | 7.14 (0.58) | 6.96 (0.64) |
|  | *16-mo.* | F | 8 (0.46) | 7.84 (0.54) | 7.82 (0.44) | 7.87 (0.5) | 7.88 (0.47) | 7.74 (0.61) |
|  |  | M | 8.09 (0.61) | 7.67 (0.8) | 7.53 (0.96) | 7.88 (0.49) | 7.87 (0.74) | 7.75 (0.77) |
| **R^2^** | *6-mo.* | F | 0.99 (0) | 0.98 (0.01) | 0.98 (0.01) | 1  (0) | 0.99  (0) | 0.98 (0.01) |
|  |  | M | 0.99 (0) | 0.99 (0.01) | 0.98 (0.01) | 1  (0) | 0.99  (0) | 0.98 (0.01) |
|  | *9-mo.* | F | 1 (0) | 0.99  (0) | 0.99  (0) | 1  (0) | 1 (0) | 0.99 (0.01) |
|  |  | M | 0.99 (0.01) | 0.99 (0.01) | 0.99 (0.01) | 1  (0) | 0.99  (0) | 0.98 (0.01) |
|  | *16-mo.* | F | 1 (0) | 0.99 (0.01) | 0.99 (0.01) | 1  (0) | 1  (0) | 0.99  (0) |
|  |  | M | 0.99 (0) | 0.99 (0.01) | 0.99 (0.01) | 1  (0) | 0.99  (0) | 0.99 (0.01) |

**Note.** F, female; M, male; C, central; Fr, frontal; Fp, frontal pole; O, occipital; P, parietal; T, temporal.

**Table S14.**

*Rhythmicity and burst properties of the participants were included in the correlation analysis between the INT and alpha band in the exploratory cohort.*

| **Parameter** | **Session** | **Sex** | **C** | **Fr** | **Fp** | **O** | **P** | **T** |
| --- | --- | --- | --- | --- | --- | --- | --- | --- |
| **Rhythm**  **Lagged**  **Coherence** | *6-mo.* | F | 0.14 (0.06) | 0.13 (0.05) | 0.13 (0.05) | 0.12 (0.06) | 0.13 (0.06) | 0.14 (0.06) |
|  |  | M | 0.15 (0.09) | 0.14 (0.07) | 0.14 (0.07) | 0.1 (0.04) | 0.14 (0.08) | 0.15 (0.08) |
|  | *9-mo.* | F | 0.19 (0.08) | 0.15 (0.07) | 0.15 (0.07) | 0.13 (0.05) | 0.16 (0.07) | 0.16 (0.07) |
|  |  | M | 0.21 (0.11) | 0.16 (0.07) | 0.16 (0.07) | 0.13 (0.06) | 0.17 (0.09) | 0.17 (0.07) |
|  | *16-mo.* | F | 0.21 (0.1) | 0.15 (0.07) | 0.15 (0.07) | 0.13 (0.08) | 0.17 (0.09) | 0.16 (0.09) |
|  |  | M | 0.21 (0.11) | 0.15 (0.07) | 0.14 (0.07) | 0.14 (0.04) | 0.16 (0.06) | 0.15 (0.06) |
| **Burst**  **Lagged**  **Coherence** | *6-mo.* | F | 0.47 (0.13) | 0.48 (0.12) | 0.47 (0.11) | 0.44 (0.11) | 0.46 (0.12) | 0.47 (0.11) |
|  |  | M | 0.47 (0.11) | 0.47 (0.1) | 0.49 (0.1) | 0.45 (0.1) | 0.46 (0.1) | 0.47 (0.1) |
|  | *9-mo.* | F | 0.43 (0.12) | 0.42 (0.12) | 0.44 (0.12) | 0.43 (0.1) | 0.43 (0.12) | 0.42 (0.12) |
|  |  | M | 0.44 (0.13) | 0.45 (0.12) | 0.46 (0.1) | 0.42 (0.1) | 0.44 (0.12) | 0.46 (0.13) |
|  | *16-mo.* | F | 0.4 (0.12) | 0.38 (0.11) | 0.39 (0.11) | 0.42 (0.09) | 0.39 (0.11) | 0.37 (0.13) |
|  |  | M | 0.36 (0.11) | 0.38 (0.09) | 0.41 (0.09) | 0.39 (0.06) | 0.34 (0.1) | 0.35 (0.11) |

**Note.** F, female; M, male; C, central; Fr, frontal; Fp, frontal pole; O, occipital; P, parietal; T, temporal.

**Table S15.**

*Rhythmicity and burst properties of the participants were included in the correlation analysis between the INT and alpha band in the validation cohort.*

| **Parameter** | **Session** | **Sex** | **C** | **Fr** | **Fp** | **O** | **P** | **T** |
| --- | --- | --- | --- | --- | --- | --- | --- | --- |
| **Rhythm**  **Lagged**  **Coherence** | *6-mo.* | F | 0.14 (0.06) | 0.13 (0.05) | 0.13 (0.05) | 0.12 (0.06) | 0.13 (0.06) | 0.14 (0.06) |
|  |  | M | 0.15 (0.09) | 0.14 (0.07) | 0.14 (0.07) | 0.1 (0.04) | 0.14 (0.08) | 0.15 (0.08) |
|  | *9-mo.* | F | 0.19 (0.08) | 0.15 (0.07) | 0.15 (0.07) | 0.13 (0.05) | 0.16 (0.07) | 0.16 (0.07) |
|  |  | M | 0.21 (0.11) | 0.16 (0.07) | 0.16 (0.07) | 0.13 (0.06) | 0.17 (0.09) | 0.17 (0.07) |
|  | *16-mo.* | F | 0.21 (0.1) | 0.15 (0.07) | 0.15 (0.07) | 0.13 (0.08) | 0.17 (0.09) | 0.16 (0.09) |
|  |  | M | 0.21 (0.11) | 0.15 (0.07) | 0.14 (0.07) | 0.14 (0.04) | 0.16 (0.06) | 0.15 (0.06) |
| **Burst**  **Lagged**  **Coherence** | *6-mo.* | F | 0.47 (0.13) | 0.48 (0.12) | 0.47 (0.11) | 0.44 (0.11) | 0.46 (0.12) | 0.47 (0.11) |
|  |  | M | 0.47 (0.11) | 0.47 (0.1) | 0.49 (0.1) | 0.45 (0.1) | 0.46 (0.1) | 0.47 (0.1) |
|  | *9-mo.* | F | 0.43 (0.12) | 0.42 (0.12) | 0.44 (0.12) | 0.43 (0.1) | 0.43 (0.12) | 0.42 (0.12) |
|  |  | M | 0.44 (0.13) | 0.45 (0.12) | 0.46 (0.1) | 0.42 (0.1) | 0.44 (0.12) | 0.46 (0.13) |
|  | *16-mo.* | F | 0.4 (0.12) | 0.38 (0.11) | 0.39 (0.11) | 0.42 (0.09) | 0.39 (0.11) | 0.37 (0.13) |
|  |  | M | 0.36 (0.11) | 0.38 (0.09) | 0.41 (0.09) | 0.39 (0.06) | 0.34 (0.1) | 0.35 (0.11) |

**Note.** F, female; M, male; C, central; Fr, frontal; Fp, frontal pole; O, occipital; P, parietal; T, temporal. The table shows the mean values (standard deviations).

**Table S16 INT_AUC – POWER AND FREQ.**

*Partial Spearman Rank correlation between tau values and alpha peak frequency and power controlling for percentage of non-convergent electrodes and fit of the models in the power spectrum decomposition. The correlation was run separately for each visit within the exploratory and validation independent samples. The table displays correlation between the alpha features and the average INT_AUC per electrode across participants, and individual’s INT_AUC across electrodes.*

| **INT calculation** | **Visit** | **n** | **Exploratory** | | **n** | **Validation** | |
| --- | --- | --- | --- | --- | --- | --- | --- |
|  |  |  | *Frequency* | *Power* |  | *Frequency* | *Power* |
| **INT per electrode (across individuals)** | *6-mo.* | - | 0.32**  [0.11 - 0.48] | -0.15  [-0.34 - 0.06] | - | 0.42***  [0.24 - 0.56] | 0.23*  [0.04 - 0.42] |
|  | *9-mo.* | - | 0.06  [-0.12 - 0.23] | -0.04  [-0.2 - 0.12] | - | 0.09  [-0.15 - 0.3] | -0.18  [-0.34 - 0] |
|  | *16-mo.* | - | 0.05  [-0.13 - 0.22] | 0.04  [-0.14 - 0.22] | - | -0.01 [-0.2 - 0.18] | 0.1 [-0.09 - 0.27] |
| **INT per individual (across electrodes)** | *6-mo.* | 43 | 0.18  [-0.16 - 0.48] | -0.01  [-0.33 - 0.32] | 39 | -0.06  [-0.41 - 0.28] | -0.21  [-0.5 - 0.12] |
|  | *9-mo.* | 37 | 0.2  [-0.14 - 0.5] | -0.49*  [-0.72 - -0.21] | 37 | -0.06  [-0.37 - 0.27] | -0.32  [-0.6 - 0.04] |
|  | *16-mo.* | 22 | -0.01  [-0.4 - 0.43] | -0.43  [-0.79 - 0.29] | 21 | -0.07  [-0.61 - 0.45] | -0.21  [-0.62 - 0.26] |

* *p* < .05, ***p* < .001, **** p* < .001. Confidence intervals were computed by bootstrapping with replacement (5000 iterations).

**Table S17 INT_AUC & LAGGED COHERENCE.**

*Partial Spearman Rank correlation between tau values and burst and rhythmic properties of the alpha controlling for percentage of non-convergent electrodes. The correlation was run separately for each visit within the exploratory and validation independent samples. The table displays the correlation between the alpha features and the average INT across individuals per electrode [Spatial Distribution] and between the alpha features and each individual participant’s INT per electrode [Individual Differences].*

| **Correlation Type** | **Visit** | **n** | **Exploratory** | | **n** | **Validation** | |
| --- | --- | --- | --- | --- | --- | --- | --- |
|  |  |  | *Burst* | *Rhythm* |  | *Burst* | *Rhythm* |
| **INT per electrode (across individuals)** | *6-mo.* | - | **0.77*****  **[0.65 - 0.84]** | 0.39***  [0.19 - 0.56] | - | **0.72*****  **[0.61 - 0.8]** | 0.3**  [0.09 - 0.5] |
|  | *9-mo.* | - | **0.69*****  **[0.58 - 0.77]** | 0.19 [-0.02 - 0.37] | - | **0.69*****  **[0.55 - 0.79]** | 0.28**  [0.09 - 0.44] |
|  | *16-mo.* | - | **0.43*****  **[0.22 - 0.61]** | 0.08 [-0.11 - 0.28] | - | **0.59*****  **[0.45 - 0.71]** | -0.14  [-0.33 - 0.08] |
| **INT per individual (across electrodes)** | *6-mo.* | 43 | 0.33 [0.02 - 0.61] | -0.03 [-0.35 - 0.29] | 39 | 0.68***  [0.45 - 0.84] | 0.03 [-0.36 - 0.42] |
|  | *9-mo.* | 37 | 0.6***  [0.14 - 0.85] | 0.2 [-0.19 - 0.48] | 37 | 0.71***  [0.5 - 0.86] | -0.23 [-0.53 - 0.17] |
|  | *16-mo.* | 22 | **0.75*****  **[0.38 - 0.9]** | -0.56*  [-0.74 - -0.17] | 21 | **0.52***  **[0.05 - 0.81]** | -0.48*  [-0.75 - -0.04] |

* *p* < .05, ***p* < .001, **** p* < .001. Confidence intervals were computed by bootstrapping with replacement (5000 iterations).
